# Impact of Increased Oxidative Stress on Cardiovascular Diseases in Women With Polycystic Ovary Syndrome

**DOI:** 10.3389/fendo.2021.614679

**Published:** 2021-02-18

**Authors:** Florentina Duică, Cezara Alina Dănilă, Andreea Elena Boboc, Panagiotis Antoniadis, Carmen Elena Condrat, Sebastian Onciul, Nicolae Suciu, Sanda Maria Creţoiu, Valentin Nicolae Varlas, Dragoş Creţoiu

**Affiliations:** ^1^ Fetal Medicine Excellence Research Center, Alessandrescu-Rusescu National Institute for Mother and Child Health, Bucharest, Romania; ^2^ Division of Molecular Diagnostics and Biotechnology, Antisel RO SRL, Bucharest, Romania; ^3^ Doctoral School of Carol Davila University of Medicine and Pharmacy, Bucharest, Romania; ^4^ Department of Cardiology, Clinical Emergency Hospital, Bucharest, Romania; ^5^ Division of Obstetrics, Gynecology and Neonatology, Carol Davila University of Medicine and Pharmacy, Bucharest, Romania; ^6^ Department of Obstetrics and Gynecology, Polizu Clinical Hospital, Alessandrescu-Rusescu National Institute for Mother and Child Health, Bucharest, Romania; ^7^ Department of Cell and Molecular Biology and Histology, Carol Davila University of Medicine and Pharmacy, Bucharest, Romania; ^8^ Department of Obstetrics and Gynecology, Filantropia Clinical Hospital, Bucharest, Romania; ^9^ Faculty of Dental Medicine, Carol Davila University of Medicine and Pharmacy, Bucharest, Romania

**Keywords:** polycystic ovary syndrome, cardiovascular disease, oxidative stress, C-reactive protein, homocysteine, miRNA

## Abstract

Polycystic ovary syndrome (PCOS) is a complex disorder that affects around 5% to 10% of women of childbearing age worldwide, making it the most common source of anovulatory infertility. PCOS is defined by increased levels of androgens, abnormal ovulation, irregular menstrual cycles, and polycystic ovarian morphology in one or both ovaries. Women suffering from this condition have also been shown to frequently associate certain cardiovascular comorbidities, including obesity, hypertension, atherosclerosis, and vascular disease. These factors gradually lead to endothelial dysfunction and coronary artery calcification, thus posing an increased risk for adverse cardiac events. Traditional markers such as C-reactive protein (CRP) and homocysteine, along with more novel ones, specifically microRNAs (miRNAs), can accurately signal the risk of cardiovascular disease (CVD) in PCOS women. Furthermore, studies have also reported that increased oxidative stress (OS) coupled with poor antioxidant status significantly add to the increased cardiovascular risk among these patients. OS additionally contributes to the modified ovarian steroidogenesis, consequently leading to hyperandrogenism and infertility. The present review is therefore aimed not only at bringing together the most significant information regarding the role of oxidative stress in promoting CVD among PCOS patients, but also at highlighting the need for determining the efficiency of antioxidant therapy in these patients.

## Introduction

Assessment of the clinical interaction between cardiovascular diseases and other interrelated pathophysiological conditions, such as polycystic ovary syndrome (PCOS), in terms of molecular and cellular changes, common biochemical and immunological pathways leading to the development of these diseases, have been intensively studied in the latest decades. To this extent, it has been shown that a variety of cardiovascular diseases (CVD) have heterogenous pathophysiologic mechanisms, where oxidative stress (OS) has been considered as one of the potential etiologies.

Under normal conditions, when the body is not subjected to a high level of oxidative stress, there is a fine balance at the physiological intracellular level of reactive oxygen species (ROS), which is maintained at low levels by various antioxidant systems. A basal concentration of ROS is essential for performing pivotal cellular functions such as gene expression or complex processes involved in signal transduction pathways (1, 2). Dysregulation of the fine balance between ROS and antioxidants at cellular level leads to the occurrence of oxidative stress that has been demonstrated to be involved in a series of pathological conditions, including cardiovascular diseases and inflammatory processes, known to be associated with a high ROS levels. Excessive ROS concentrations act on cell macromolecules by promoting cell necrosis and apoptosis, thus affecting the normal course of multiple cellular functions (1, 3–6).

With regard to the female reproductive tract, although ROS indeed play certain physiological roles, including the modulation of several functions such as ovarian steroidogenesis, corpus luteal function and luteal regression, fertilization, and the development of the early embryo, numerous studies have demonstrated the pathological effects of these molecules, involved in a multitude of diseases (7). Further on, in relation to the mechanisms by which oxidative stress affects the cardiac function at cellular level, it has been shown that the occurrence of hypertension may be due to the process of vasoconstriction that takes place as a result of a decreased availability of nitric oxide due to increased ROS levels, concentrations which further impact the cardiac function by negatively influencing calcium signals, thus leading to arrhythmia. Additionally, it has been speculated that the increase in ROS levels could also influence cardiac remodeling and atherosclerotic plaque formation (1, 8). Although several studies have evaluated the correlation between cardiovascular diseases and PCOS, the association of this syndrome with subclinical and/or clinical forms of cardiovascular disease, independent of the risk factors common to the two diseases, the exact interrelationship between these conditions has not been clearly elucidated.

PCOS is a disease that presents heterogeneous clinical variants, in which the pathogenesis involves the existence of several cardiometabolic abnormalities such as metabolic syndrome, glucose intolerance, dyslipidemia, hypertension, diabetes, all of which are also risk factors for CVD diseases (9, 10). Furthermore, PCOS is characterized by polycystic ovarian morphology that leads to ovarian dysfunction such oligo- or anovulation, where the central neuroendocrine systems perform an important role, due to excessive luteinizing hormone (LH) and gonadotropin-releasing hormone (GnRH) levels and relative follicle-stimulating hormone (FSH) deficiency, that contribute to the ovarian hyperandrogenemia and altered folliculogenesis, characteristic features of PCOS (11–13).

PCOS is a heterogenous syndrome that manifests through changes in the metabolic balance in which mitochondrial dysfunctions have been shown to facilitate the progression and occurrence of various complications of this disease (13). Although the etiology and pathophysiology of PCOS are not yet fully elucidated, it is currently considered that the main pathophysiological mechanism leading to this syndrome is the excess of androgen hormones, which results in metabolic, reproductive, and not least cosmetic changes, consisting of an increased body mass index due to a predisposition to obesity, as well as changes in the appearance of the skin due to acne outbreaks (12–15). Moreover, recent studies have highlighted the link between the pathogenesis of PCOS and chronic inflammatory status, with published data showing that numerous inflammatory markers are elevated in women suffering from PCOS (13, 16, 17). An additional possible cause of PCOS has been shown to be oxidative stress that could cause genetic changes such as point mutations, DNA strand breaks, aberrant DNA cross-linking, DNA-protein cross-linking, and DNA methylation, ultimately leading to the silencing of certain tumor suppressor genes (18–22).

### PCOS—Definition

PCOS is a heterogeneous ailment described in women of childbearing age, characterized by ovulatory dysfunction, androgen excess, and polycystic ovarian morphologic features (23, 24). Also known as the Stein-Leventhal syndrome, it is a common endocrinopathy among women of reproductive age. PCOS affects 6% to 15% of women at the reproductive age, depending on diagnostic criteria (25, 26). The Rotterdam criteria (2013) are the most commonly used criteria to diagnose PCOS, and include the following: ovulation disorder, hyperandrogenism diagnosed by biochemical testing and/or clinical aspects, and ovarian volume over 10 ml or 12 or more ovarian cysts. The diagnosis can be established when two of the three conditions are fulfilled (27). Based on these criteria, four PCOS phenotypes can be detected, namely ovulation disorders, polycystic ovary, and hyperandrogenism, making up the classic phenotype, normal ovarian ultrasonography with hyperandrogenism and ovulation disorder, polycystic ovary ultrasonography image and hyperandrogenism, with no ovulation abnormalities, and no evidence of hyperandrogenism, but with polycystic ovary ultrasonography image and ovulation disorders (28, 29). Several endocrinopathies can mimic PCOS, such as Cushing’s syndrome, non-classic adrenal hyperplasia, drug-induced androgen excess, and androgen-producing tumors (30). Ovulatory dysfunction can further be found in conditions like hyperprolactinemia or thyroid dysfunction (31). Therefore, in order to proper diagnose PCOS, these disorders need to be excluded.

PCOS is characterized by the overproduction of ovarian androgen hormones, especially testosterone, as a result of an excessive production of LH in the pituitary gland or due to hyperinsulinemia, if the ovaries are sensitive to insulin. Common symptoms noticed in women with PCOS are infertility, signs of androgen excess such as hirsutism, virilization, acne, alopecia, and menstrual irregularities, including amenorrhea and dysfunctional bleeding (32). Women with PCOS also have an increased prevalence of certain comorbidities, such as dyslipidemia, excess weight, metabolic syndrome, type 2 diabetes, and hypertension. Along with other features such as chronic low-grade inflammatory state and endothelial dysfunction, PCOS poses an elevated risk of developing cardiovascular disorders (33).

### PCOS—Pathophysiology

There are many hypotheses regarding the pathophysiology of PCOS, including among them ovarian hyperandrogenism, follicles resistant to rupture due to shell thickness, hypersecretion of luteinizing hormone, increased anti-Mullerian hormone (AMH), which is a blocker paracrine factor for follicular development, and hyperinsulinemia (34). These abnormalities can appear due to hormonal, metabolic, or even toxic factors occurring during the embryonic stage and/or in the early development of the female gonad, or because of certain epigenetic changes (35). The genetic basis of PCOS is suspected on the grounds of the aggregation of this syndrome in families, since it has been shown that within first-degree relatives, about 20 to 40% of women also have the disorder (23, 34, 36, 37).

#### Gonadotropins

Gonadotropin-releasing hormone (GnRH) neuropeptides released from neurons into the portal vein and median eminence stimulate the adenohypophysis gland to secrete gonadotropins, which mediate ovarian steroidogenesis and folliculogenesis. The follicle-stimulating hormone (FSH) binds to FSH receptor on the granulosa cells and stimulates follicular maturation and ovulation (38). On the other hand, the luteinizing hormone (LH) stimulates steroidogenesis, follicular growth, and corpus luteum formation (39, 40). Anovulation is determined by inappropriate gonadotropin secretion. Specifically, modified pulsatility of GnRH consisting of elevations in the amplitude and frequency of secretion, generates an increased production of LH compared to that of FSH. It is unknown whether hypothalamic dysfunction is a determining cause of PCOS or is caused by an abnormal steroid feedback. In both cases, the level of LH is reported to be high, while the LH/FSH ratio is increased to over 2/1 (36).

The impact of peripheral hormones on the brain function in the pathogenesis of PCOS has been explained through four suggested hypotheses. The first hypothesis is based on the negative feedback of steroid hormones which appears after setting up changes of the critical neuronal circuits determined by hyperandrogenism (40). The second hypothesis revolves around the hyperinsulinemia that stimulates the activity of GnRH neurons and the response of the pituitary gland to GnRH (41). The third hypothesis refers to the low concentration of progesterone in serum that is followed, in PCOS, by anovulation, which eventually eliminates the influence of the progesterone negative feedback on the release of GnRH (42). The fourth hypothesis states the function of the pulse generator of GnRH that reduces the activity of GnRH inhibitors (40, 43). Overall, the hypothalamic-pituitary-gonadal axis remains one of the principal regulators of female reproduction, its dysfunction leading to ovulation disorders.

#### Hyperandrogenism

**Figure 1:**
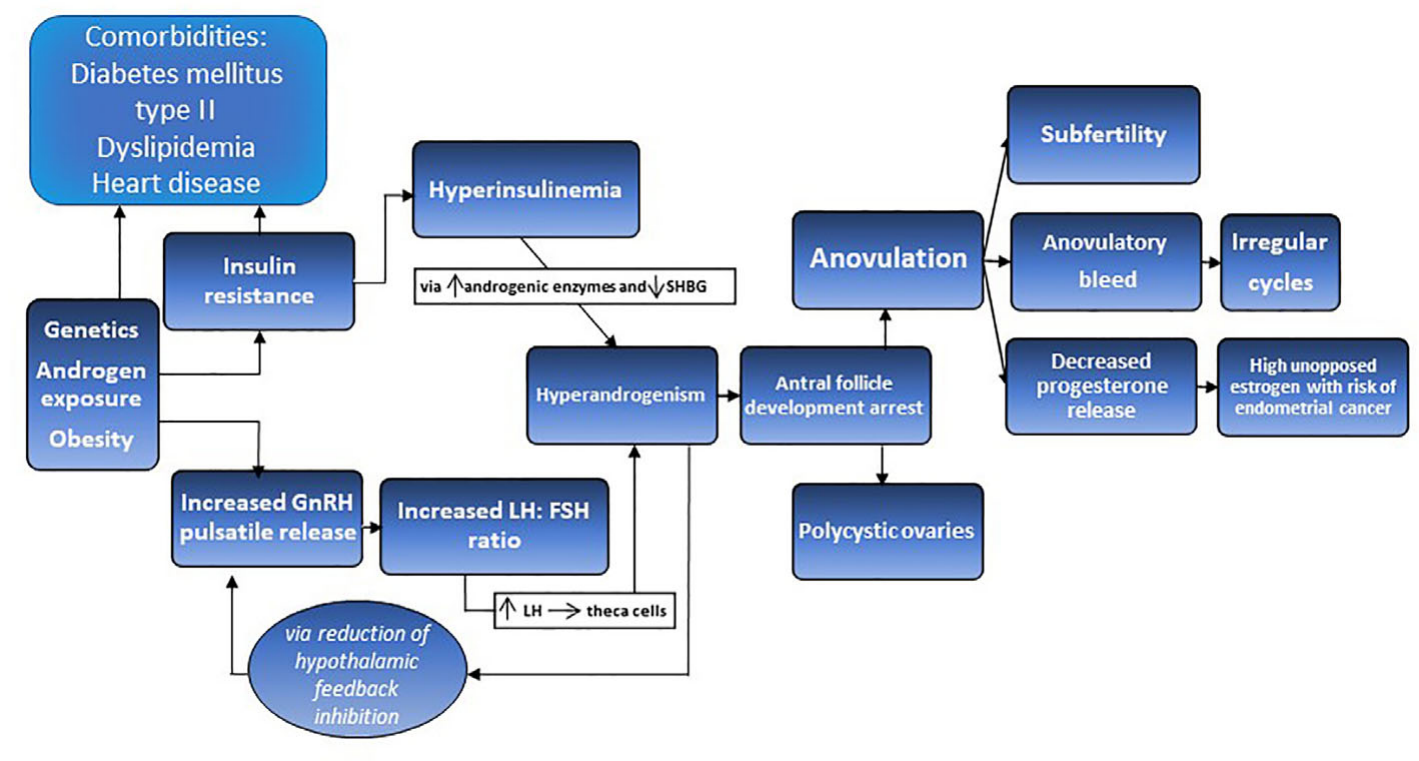
The proposed pathophysiology of PCOS is a synergistic relationship between perturbed gonadotrophin releasing hormones (GnRH) pulsatility and insulin resistance, accompanied by hyperinsulinemia and hyperandrogenism leading to antral follicle development arrest, anovulation, irregulate cycles, subfertility, and polycystic ovaries.

A fundamental characteristic of PCOS is the increased production of androgens in ovaries, due to excessive activity in the theca cells stimulated by intraovarian or extraovarian factors (44). LH and insulin stimulate the production of androgens, determining elevated levels of dehydroepiandrosterone (DHEAS) and testosterone (36) (**Figure 1**). High levels of free testosterone were noticed in about 70% to 80% of patients with PCOS, while 25% to 65% expressed elevated DHEAS levels. This leads to increased estrone levels by peripheral conversion mechanism, which converts androgens to estrogens using aromatase. Furthermore, low levels of sex hormone-binding globulin (SHBG) were reported in women diagnosed with PCOS (36). Liver synthesis of SHBG is reduced by insulin as well as progestins, androgens, corticoids, and growth hormones (45). Reduced SHBG production leads to lower levels of bound circulating androgens, thus resulting in more available androgens capable of binding to organ receptors. Consequently, clinical hyperandrogenism is determined by high levels of free testosterone, although total testosterone might be within the normal range (46).

Exposure to androgens throughout fetal development has been speculated as another reason for hyperandrogenism determining the phenotypes of PCOS in adulthood (47). In this regard, there are four hypotheses for the exposure to additional androgens during the embryo stage. Firstly, the evolution of the hypothalamic-pituitary axis simultaneously with certain hypothalamic-pituitary axis disorders in embryonic development are thought to increase the production of androgen hormones (48). Secondly, in mothers with PCOS, the placenta is limitedly capable of aromatization and increasing of SHBG concentration, thus causing the fetus to receive maternal androgens through the placenta (49). The third hypothesis suggests a fatal genetic disorder with undifferentiated ovaries that can be the source of increased androgen production (35). The fourth hypothesis refers to malformations that increase the androgen production, such as hyperplasia of the adrenal glands (50). Either way, in order to diagnose PCOS in women, one must look at the biochemical androgen profile, which includes free and total testosterone, SHBG, DHEAS, 17-hydroxy-progesterone and the free androgen index (FAI), estimated as the total testosterone level divided by SHBG and multiplied by 100 (51).

The steroidogenic cells of the adrenal cortex and the ovary stand at the origin of the hyperandrogenemia that characterizes PCOS, using similar enzymes for steroidogenesis (52). The Cytochrome P450 Family 19 Subfamily A Member 1 (CYP19A1) gene encodes the aromatase, enzyme which turns androgens into estrogens. In the ovarian follicles, reducing the activity of aromatase leads to hyperandrogenism, and a positive correlation between the incidence of PCOS and mutations in this gene has been observed (53). Furthermore, an androgen excess has been indicated to determine hypertension by stimulating the expression of adipose tissue aromatase (54, 55).

#### Hyperinsulinemia

Insulin is the hormone primarily responsible for lipogenesis and glucose homeostasis. Insulin has effects on fat, protein metabolism, carbohydrates, while also acting as a mitogenic hormone (56). The ovary and adrenal cortex are steroidogenic tissues in which insulin promotes steroidogenesis by potentiating the cognate trophic hormones (57). Insulin resistance associated with compensatory hyperinsulinemia determines excessive adrenal and/or ovarian androgen secretion and decreases the synthesis of SHBG in the liver, thus resulting in an increase of circulating testosterone concentration. Intrinsic insulin resistance is characteristic of women with PCOS independent of the magnitude of androgen levels and extent of obesity, with lean PCOS patients also experiencing it (28). Insulin resistance leads to reduced glucose-uptake response in spite of high insulin levels. This is the result of decreased insulin sensitivity due to abnormal signal transduction at receptor and post-binding level (36).

Alternate theories emphasize the fact that LH levels negatively correlate with insulin levels in women, an aspect demonstrated experimentally in both normal and PCOS women under euglycemic/hyperinsulinemic clamps (58, 59). Loss of negative feedback in the hypothalamus elevates LH, which may drive increased androgen production, but it is androgen that results in insulin resistance (60, 61). Elevated androgen levels positively correlate with LH levels, suggesting a failed compensatory mechanism prompting elevated LH output. Thus, loss of negative feedback in the hypothalamus can lead to both PCOS and increased heart disease, which may also be aggravated by increased obesity (62). The paradox of insulin signaling witnessed in PCOS is that the adipose tissue, liver, and skeletal muscles exhibit insulin resistance, whereas the pituitary and steroid-producing tissues retain insulin sensitivity. This aspect has been illustrated by observing the different actions of insulin in granulosa lutein cells from patients with PCOS and anovulation (28). In women with PCOS, the prevalence of metabolic syndrome is approximately threefold higher and is defined as the association of hyperglycemia, obesity, dyslipidemia, and hypertension (63). However, the definition of metabolic syndrome is incomplete in adolescents, being characterized by a combination of low high-density lipoprotein (HDL) cholesterol levels, high triglyceride concentrations, increased waist circumference, elevated fasting blood glucose, and hypertension for age (28, 64, 65).

#### Ovaries

Ovulation results from coordinated signaling by the hypothalamus-pituitary axis, ovarian granulosa cells, ovarian theca cells, and the developing follicle (66). In women with PCOS, this process malfunctions because of the abnormal development and failure in selecting a dominant follicle, thus inducing anovulation (67). The ovulatory dysfunction is characterized by increased activation of the follicles, followed by arrested growth before the maturation of these follicles can occur. Furthermore, PCOS follicles also have lower rates of atresia, which may explain why premature depletion of the follicular pools seldom occurs in the ovaries of these women (68). Due to anovulation, progesterone is lacking, thus leading to chronic estrogen exposure. This has an impact on the endometrium by constant mitogenic stimulation with endometrial thickening which leads to unpredictable bleeding or endometrial cancer (69).

In normal folliculogenesis, growth factors such as growth differentiation factor 9 (GDF-9) and bone morphogenetic protein 15 (BMP15), also referred to as oocyte-secreted growth factors (OSFs), aid in the development from primordial to primary stage follicles, while subsequent stages, up to the selection of the dominant follicle are regulated by FSH to (70). Throughout folliculogenesis, insulin and androgens have a synergistic aspect with LH, which exerts its effect from the middle to the late follicular stage (71). The equivalence between AMH and FSH may play a primary role in the aromatase activity, both during and after dominant follicle selection. Moreover, increased estradiol emission by the dominant follicle suppresses FSH levels, leading to subordinate follicle dissolution resulting in mono-ovulation (72). Under excessive androgen exposure, accelerated early follicular growth in PCOS tends to take place, leading to small-follicle occurrence. Decreased OSFs levels further lead to intensified early folliculogenesis (73). Further on, small follicle excess promotes high AMH levels, which in turn mediate follicle responsiveness to FSH (74). To this extent, low FSH responsiveness and premature granulosa cell luteinization denature the dominant follicle selection, producing follicular arrest (75). High insulin levels can further induce premature luteinization along with LH receptor expression (76).

Follicular defects associated with PCOS are defined by early and accelerated follicular growth as well as distortion in the subsequent stages in relation to dominant follicle selection, leading to follicular arrest (77). In this regard, Webber et al. have reported a greater density of small preantral, especially primary follicles in analyzed ovarian biopsies belonging to women diagnosed with PCOS in comparison with control groups (78). Atresia deceleration, later demonstrated by the same team of researchers, may answer for the increased recruitment and explain why premature follicle depletion does not occur in polycystic ovary (79). Arrested follicle development in women with PCOS can be explained by the relatively low levels of circulating FSH, which hinder the normal maturation process (80). Additionally, LH hypersecretion is detrimental to ovulation and follicular growth, since it determines decreasing FSH sensitivity, thus contributing to the premature luteinization of granulosa cells (32).

Anovulation can also be determined by altered GnRH pulsatility and improper gonadotropin secretion, both leading to menstrual irregularity (81). Moreover, anovulation can also be facilitated by insulin resistance, as many anovulatory patients diagnosed with PCOS express ovulatory cycles after treatment with insulin sensitizers such as metformin (82, 83). Increased intraovarian androgens from large antral follicles may also cause anovulation in patients with PCOS, fact which is supported by the improvement of menstrual regularity in patients who underwent laparoscopic ovarian drilling or ovarian wedge resection (36).

#### Inflammation

Low-grade systemic inflammation associated with PCOS is indicated by the high levels of inflammatory markers such as interleukin-18 (IL-18), C-reactive protein (CRP), white blood count, and monocyte chemoattractant protein-1 (MCP-1), along with increased oxidative stress and endothelial dysfunction (84). These inflammatory markers stimulate the proliferation of theca cells, while also promoting steroidogenesis, and contributing to follicular atresia and hyperandrogenemia (13).

Hyperglycemia further plays a role in PCOS-related inflammation, due to mononuclear cells utilizing glucose as a redox substrate, thus leading to high levels of ROS and inducing oxidative stress (85). ROS production by immune cells as a result of oxidative stress plays a primordial role in both the development and progression of endothelial dysfunction, which significantly contributes to the occurrence of arterial hypertension along with other cardiovascular diseases. Furthermore, insulin resistance and chronic inflammation play important roles in the etiopathogenesis of diabetes mellitus type II and metabolic syndrome, common comorbidities among PCOS women (33, 86).

## Cardiovascular Disease in PCOS

**Figure 2:**
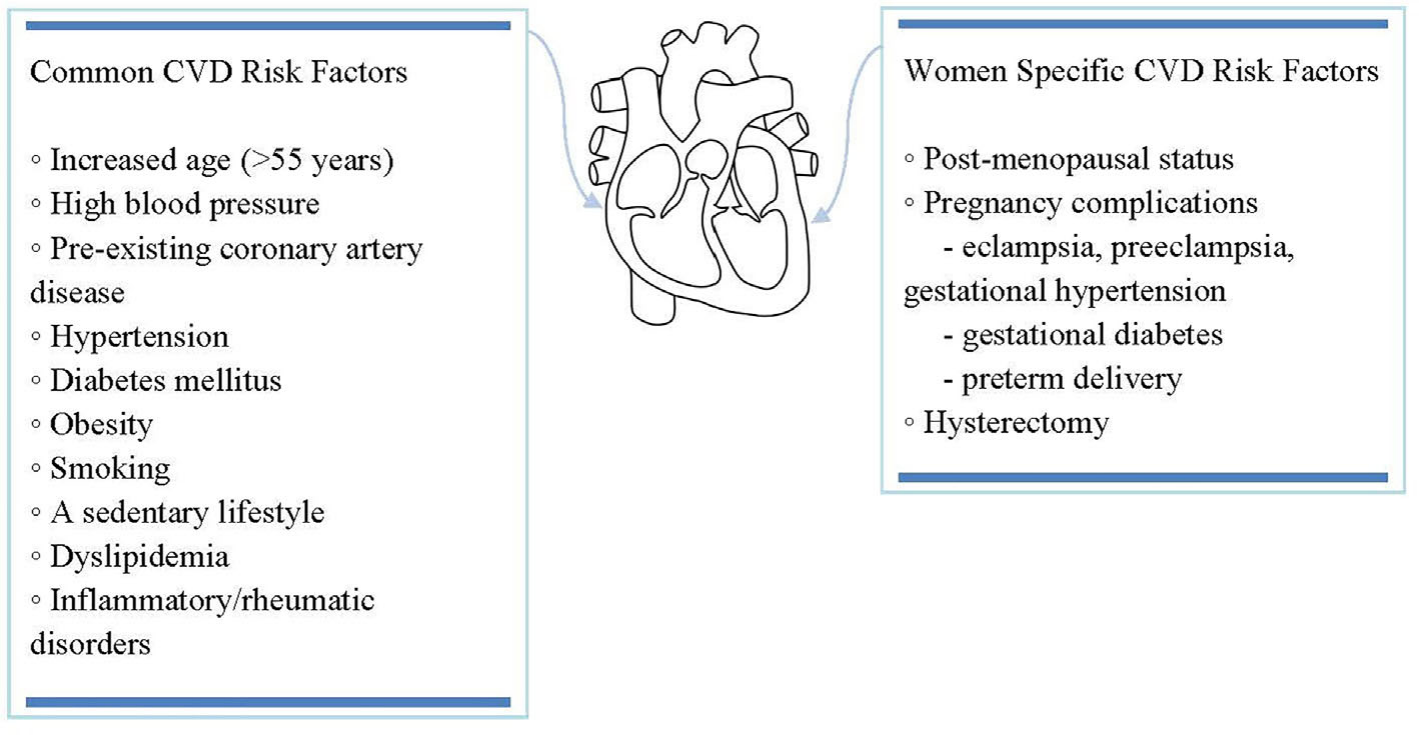
While most cardiovascular risk factors in women overlap with those in men, several circumstances remain characteristic of women.

While significant improvement in the incidence and general outcome of cardiovascular diseases has been observed in the past decades, they go on being the leading cause of death among women worldwide (87, 88). Furthermore, preventive care including counseling and prophylactic treatment is less likely to be offered to women than men with similar atherosclerotic cardiovascular disease risk (87, 89), while medical management of these patients tends to be less vigorous, thus more rarely achieving optimal results (90, 91). While most cardiovascular risk factors in women overlap with those in men, several circumstances remain characteristic of women (92, 93), as it can be seen outlined in **Figure 2**.

Cardio-metabolic disturbances have been found in women with PCOS regardless of age, posing significant risks for the occurrence of CVD. These disturbances are represented primarily by atherogenic dyslipidemia, hypertension, obesity, along with insulin resistance, impaired glucose tolerance and type II diabetes (94, 95). The association between PCOS and CVD has been related to this partial overlapping of risk factors. While PCOS is influenced by race, BMI and age, with symptoms becoming less thunderous with increasing age and most of them disappearing after the onset of menopause, cardio-metabolic disorders can, however, continue to pose a threat to the patients’ health (94, 96–99). If earlier studies regarding the higher risk of CVD in women with PCOS could not establish its absoluteness (100), more recent data confirm that the metabolic dysfunction typical of women with PCOS leads to a definite increase in CVD events (101–103).

### Hypertension

The pathophysiology of hypertension in PCOS is multifactorial, depending on factors such as obesity, hyperandrogenism, elevated sympathetic nervous system activity, and insulin resistance (104). Several studies indicated that patients with PCOS are more likely to develop hypertension as opposed to the normal population. However, this fact is somewhat unclear, since PCOS is associated with obesity as well, which also represents a significant risk factor for hypertension. Therefore, the interpretation of these studies is rather complicated, since obesity is a variable not usually considered in many types of research (105–111). Still, a meta-analysis performed by Amiri et al. showed that hypertension is more common in women with PCOS than in the control population. Moreover, they have separately evaluated women during post-menopause and reproductive-age women with PCOS because, since it is well known that the prevalence of hypertension is higher with aging and with menopause onset. The result was that, even after adjusting diabetes mellitus and BMI variables, PCOS women during reproductive age were more likely to develop hypertension (112).

As mentioned previously, a significant risk factor for hypertension is represented by obesity. In this regard, it has been shown that the prevalence of obesity and overweight status among PCOS patients is 80% higher compared with non-PCOS women, with PCOS women associating BMIs over 30 kg/m^2^ and higher waist-hip ratios (113), more commonly in Caucasian than Asian women (114). Obesity in females suffering from PCOS may be correlated with insulin resistance, which generates hyperinsulinemia that triggers ovarian steroidogenesis. This way, sex hormone-binding globulin production is downregulated and, as a consequence, the availability of free androgens is elevated, causing visceral accumulation of fat, thus facilitating central obesity (113–115). In PCOS patients, it was observed that a combination of factors like insulin resistance, obesity, and hyperandrogenism leads to an elevated sympathetic nervous system activity, each factor being a possible mediator of hypertension (116–118).

Deficiencies in the hypothalamic-pituitary axis produce an excessive secretion of LH and a low excretion of FSH, hormonal imbalance that leads to secretory changes in the inner sheath of ovarian follicles. In turn, an excess of androgenic hormones is released, which is responsible for both clinical and paraclinical signs of hyperandrogenism (119, 120). Numerous women with PCOS, especially those with hyperandrogenic phenotype, have various cardio-metabolic disturbances that increase the risk of developing hypertension (121, 122). One study demonstrated that, with age, almost half of women with PCOS improve due to the decrease of serum androgens as a consequence of adrenal and ovarian aging (123). Testosterone levels drop with age in both PCOS and healthy women, the decrease being observed years before the onset of menopause (124). This fact may lead to a progressive reduction of CVD risk factors (125). However, the processes that determine the lowering of hypertension risk remain slightly vague. An analysis of daytime ambulatory blood pressure revealed that young and obese women suffering from PCOS had elevated blood pressure in comparison with non-PCOS females (107). Other variables that must be taken into consideration are background aspects of the individual such as ethnicity and race. To this extent, Lo et al. revealed that, even after adjusting for diabetes mellitus, age, and BMI, the prevalence of hypertension and/or high blood pressure was increased in black women with PCOS when compared to the Caucasian population, and among the latter, Hispanic and Asian women were the least affected (126).

### Atherosclerosis and Vascular Disease

Dyslipidemia is a cardio-metabolic disturbance distinguished by high levels of LDL cholesterol and triglycerides and low levels of HDL cholesterol, found in both obese and lean women with PCOS (127, 128). This imbalance, together with obesity and insulin resistance, predisposes these females to a subclinical vascular disease characterized by intimal-medial thickening in the carotid arteries, coronary artery calcifications, and endothelial dysfunction (129–131). These modifications could put PCOS patients at risk for developing cardiac events, both fatal and nonfatal, as well as strokes (132).

Carotid intima-media wall thickness (cIMT) is a determination of the tunica media and tunica intima of the arteries, evaluated usually by ultrasound performed on large vessels close to the skin, as is the carotid artery. This measurement is utilized for the detection of atherosclerosis and for tracking its regression or progression, and it is correlated with the prevalence of myocardial infarction or stroke (133–139). cIMT is known to be associated with visceral adiposity, dyslipidemia, hyperinsulinemia, and raised systolic blood pressure, risk factors also encountered in PCOS (140–146). Meyer et al. performed a meta-analysis that showed that cIMT is elevated in females with PCOS compared with the control group, suggesting an elevated risk for accelerated atherosclerosis in PCOS patients (147). Talbott et al. further demonstrated that increased cIMT was noticed in females ≥ 45 years, explaining that CVDs have long incubation periods, with metabolic disturbances occurring in young age converting into carotid damage by older age, and it seems that cIMT is more affected by the combination of age and PCOS than by aging alone (131).

The severity of coronary atherosclerosis is indicated by the coronary artery calcium (CAC) score, an independent risk marker for sudden cardiac death and myocardial infarction in both symptomatic and asymptomatic patients (148), with several studies focusing on elevated CAC scores in PCOS patients. For instance, Christian et al. performed a study that included premenopausal women at 30 to 45 years old suffering from PCOS and found a higher prevalence of elevated CAC scores in PCOS women than in the control group (149). Another study performed by Talbott et al. reported that elevated CAC had a higher prevalence among PCOS females between 40 and 61 years old (46%) than the control group (31%), even after controlling for BMI and age (150). Shroff et al. further conducted a research study designed to discover early-onset increased CAC score as an indicator of subclinical atherosclerosis in young and obese PCOS females (151). Compared with weight and age-matched controls, early coronary atherosclerosis was detected in young females suffering from PCOS. Due to the young age, the subjects in the study did not associate other CVD risk factors, therefore PCOS was speculated to contribute to the risk of elevated CAC scores (151).

It is broadly accepted that chronic inflammation is correlated with endothelial dysfunction. Abnormal morphology, disposition, and function of the adipose tissue in PCOS females are correlated with the generation of chemokines, cytokines, and low-grade inflammation, which lead to the activation of hypoxia-induced pathways, with the consequential reduction of adiponectin production (152). This pro-inflammatory condition is correlated with the progression of insulin resistance, thus promoting type II diabetes development along with increasing cardiovascular risk (153). Furthermore, it is presumed that androgens are mediators in the transformation of preadipocytes into mature adipocytes, while also having an impact on oxidative stress, lipid, and glucose metabolism (99, 154). Overall, females with PCOS, due to their underlying pathophysiology, could be at risk for cardiac and cerebrovascular disease. Contrasting results that were obtained during several studies prompted more research, especially in the form of longitudinal studies, focusing on cardiovascular assessment and follow-up of these women for a better understanding and management of PCOS complications.

## Oxidative Stress in PCOS and CVD

At a biological level, oxidative stress refers to the physiological disturbances between free radical species such as ROS or reactive nitrogen species (RNS), and the body’s ability to eliminate them. Oxidative stress can also be defined as the discrepancy between signaling systems and redox control systems (155–157). Living organisms have developed several mechanisms to respond to oxidative stress by producing antioxidants. A change in the balance between oxidizing and antioxidant substances in favor of excess oxidants leads to oxidative stress. These systems include enzymes (superoxide dismutase, catalase, and glutathione peroxidase), antioxidant macromolecules (albumin, ceruloplasmin, and ferritin), antioxidant micro-molecules (ascorbic acid, α-tocopherol, β-carotene, ubiquinone, flavonoids and glutathione, methionine, uric acid) bilirubin (1, 158–161).

ROS are highly reactive molecules with a very short lifespan, and are classified into two categories, namely non-radical species, including hydrogen peroxide (H2O2), hypochlorous acid (HOCl-), ozone (O3-), lipid peroxides (LOOH), along with hydroperoxides (ROOH), and radical species, consisting of superoxide anion (O2•−), singlet oxygen (1O2), hydroperoxyl radical (HOO•), hydroxyl radical (•OH), with •OH being considered the most important ROS (158). RNS on the other hand comprise a range of various chemical compounds derived from nitric oxide (NO) in the reaction of biologically generated free radicals that tend to form more stable species, a process that generates multiple biological effects (162). Free radical species are extremely unstable molecules that tend to gain stability by acquiring electrons from neighboring molecules such as nucleic acids, carbohydrates, proteins, and lipids, which leads to a cascade of chain reactions, that cause cell damage (155, 163–167). Free radicals fulfilling important roles in physiological and pathological conditions, come from both endogenous and exogenous sources. They are the result of cellular processes such as oxygen reduction through the electron transport chain in the mitochondria, but could also be generated in the endoplasmic reticulum, phagocytic cells, peroxisomes, as well as other cell compartments, as a result of central processes such as protein phosphorylation and activation of certain factors specific for transcription, apoptosis, and immunity (168).

When the body’s ability to eliminate excessive ROS and/or RNS molecules is exceeded, and they remain in the intercellular space for longer periods of time, oxidation of sensitive biomolecules takes place, such as lipid peroxidation (LPO), essential fatty acid oxidation, or oxidation of guanine DNA-base, causing damage to proper cellular function (169). Among the cellular components involved in regulating OS levels, mitochondria play an important role, dysfunctions at this level having been demonstrated to assist in the pathogenesis of several diseases, including PCOS, metabolic syndrome and diabetes mellitus, cardiovascular disease, and cancer (13, 170, 171). Thus, mitochondrial dysfunction in combination with systemic inflammation is thought to play an essential role in the occurrence of complications associated with metabolic disorders in patients with PCOS, and in the predisposition to cardiovascular disease. In this regard, as a result of mitochondrial dysfunctions, systemic increase of OS occurs in patients of reproductive age who develop symptoms of PCOS, and who have been found to have elevated serum levels of inflammatory markers such as C-reactive protein, interleukins, and proinflammatory cytokines, increased cell counts of leukocyte series such as lymphocytes and monocytes, change in tumor necrosis factor (TNF-α), as well as increases in some metabolites resulting from the processes of carbonylation and oxidation of proteins and lipids (172–174).

Previous studies have revealed that women with PCOS, due to their altered lipid profile, may present certain dysregulated markers, such as increased body mass index, triglycerides, total cholesterol and LDL levels, along with decreased total HDL and HDL2 levels (175–177). In this regard, in PCOS women, several specific metabolites such as nitric oxide (NO) and malondialdehyde (MDA), resulting after lipid metabolization through the reduction of mono- and polyunsaturated fatty acids (MUFAs and PUFAs) and considered oxidative stress markers, have been found at higher levels when compared to control lots (178). On the other hand, Sulaiman et al. have demonstrated the decreased levels of antioxidant molecules glutathione (GSH) and total antioxidant capacity (TAC), capable of cancelling out the destructive impact of free radicals (179). Furthermore, it has been postulated that, especially among women with PCOS, dietary factors may accomplish an important role in promoting the metabolic imbalance (177). For instance, Kazemi et al. have evaluated the relationship between four dietary patterns and the overall ovarian function, and found that the latter was affected by diets that influence obesity, metabolic status and hyperandrogenism regulation (176). The Dietary Approaches to Stop Hypertension (DASH) eating plan has also been previously analyzed by Asemi and colleagues, who highlighted the effect of the DASH diet not only on lipid profiles, but also on oxidative stress markers in PCOS women. They found that women with PCOS undergoing the DASH diet could register significant reduction in insulin, triglyceride and very low-density lipoprotein cholesterol (VLDL-C) values, along with an increased capacity of prooxidant status by elevated levels of total antioxidant capacity (TAC) and GSH (175).

The etiology and circumstances that define the severity of PCOS and the occurrence of risk factors in the development of cardiovascular disease involve the endothelial dysfunction caused by an imbalance between the production and bioavailability of vasoactive molecules that either contract or relax the vessel. Molecules such as endothelium-dependent relaxing factors, endothelium-dependent hyperpolarization factors, endothelium-dependent constricting factors, vasodilator prostaglandins, nitric oxide (NO), fulfill an important role in maintaining a balance for tissue oxygen needs, while also being involved in central processes such as the remodeling of vascular structures by adjusting the vascular tone and diameter to adapt to the metabolic demand in every particular situation (1, 8, 180, 181).

## CVD Markers in PCOS

In light of the absence of conventional CVD risk factors in PCOS women, various studies have focused on the relevance of subclinical CVD markers among these patients. In this regard, CRP and homocysteine have consistently been shown to be increased in the plasma of patients with PCOS. At the same time, emerging microRNA (miRNA) analysis methods have enabled the identification of various dysregulated miRNAs, as a response to metabolic changes characteristic of this condition.

### C-Reactive Protein (CRP)

CRP is a very common circulating marker, that is usually used as an inflammatory index for individuals. Recent studies have demonstrated the inducing function of CRP in inflammation, as the protein promotes the activation of the complement pathway, induces apoptosis, phagocytosis, and the production of proinflammatory cytokines, such as IL-6 and TNF-α (182). The fact that CRP has been observed to be increased in women with PCOS implicates chronic inflammation as a mechanism that contributes to the increased risk of CVD in women with PCOS (183). A large study performed in 2011 compared CRP levels in the serum of 2.359 women with PCOS with those from 1.289 healthy women, pointing out the significant difference between the two groups, as the group with PCOS had a mean value 95% higher than the control group (184). These findings were irrespective of the high body mass index (BMI), as they had not changed much after eliminating the bias from BMI.

Different approaches have been indicated to be beneficial for the reduction of CRP in women with PCOS, such as medication with statins or an increase in daily activity. In a study carried out in 2008, which included 40 medication naïve women with PCOS, an effective reduction of mean high sensitivity CRP (hs-CRP) in serum was demonstrated after 12 weeks of atorvastatin administration. This reduction was around 1.5 mg/liter and was accompanied by a reduction of mean levels of total cholesterol, LDL cholesterol, triglycerides, testosterone, and insulin resistance (185). Moreover, in another study, an increase of 1000 steps per day was associated with a decrease of 13% in serum CRP levels for a group of 65 women with PCOS, following 6 months of increased daily activity. For this research, data was adjusted for different parameters, such as age and baseline step count, while the observed reduction in CRP levels had a p-value of 0.005 (186).

### Homocysteine

Homocysteine is a well-known marker of oxidative stress, as it has the ability to promote the production of ROS, and, when in high concentration, it can induce the injury of endothelial cells (187). In a big meta-analysis performed in 2013, a group of 4.933 women with PCOS has been compared with a control group of 3.671 healthy women for the detection of circulating markers that indicate OS and PCOS (188). The findings of this study pointed out a 23% higher mean concentration of homocysteine in the group of women with PCOS, implying the increased levels of OS in this group. Homocysteine can induce OS and increase the risk of CVD in PCOS patients by restricting the expression and the activity of glutathione peroxidase and superoxide dismutase (SOD), while promoting the expression of inducible nitric oxide synthase (iNOS). Moreover, it induces the expression of NADPH oxidase and diminishes thioredoxin, thus favoring the build-up of ROS (189).

The implication of homocysteine for the development of CVD has been noted since the 1990s, due to the promotion of atherosclerosis and hypercoagulability (190). Apart from PCOS patients, homocysteine has been associated with CVD, such as coronary artery disease (CAD), in individuals with chronic renal dysfunction (191). The fact that atherosclerosis is a pathological process with very strong associations with the onset of CVD, correlates hyperhomocysteinemia with conditions such as stroke, heart failure, and myocardial infarction (192). Moreover, there has been described a strong correlation between homocysteine and CRP expression in vascular smooth muscle cells (VSMCs). In this regard, it has been shown that increased levels of homocysteine can induce the expression of CRP at the transcriptional and the translational level, through harnessing signal pathways of N-methyl-D-aspartate receptor (NMDAr) in VSMCs (193). Therefore, a connection between hyperhomocysteinemia and inflammation comes up, which further corroborates the role of homocysteine in atherosclerosis.

The correlation of homocysteine with CAD has also been pointed out in a study where 70 patients were monitored and compared for their homocysteine serum levels and the presence of CAD through coronary angiography. The patients with CAD had considerably higher levels of homocysteine at a fasting state compared to individuals without CAD, showing increased statistical significance (p < 0.001) (190). In addition, the severity of CAD has been found to be associated with the levels of homocysteine, having a p-value below 0.001. Homocysteine seems to induce the proliferation of VSMCs while also augmenting the activity of HMG Co-A reductase, which promotes the synthetic production of cholesterol (190). These findings highlight once again the significant role of homocysteine in atherosclerosis.

Over and above, homocysteine has been implicated in the progress of increased arterial stiffness, as it has been correlated with increased aortic stiffness and pulse pressure. Although the mechanism that connects hyperhomocysteinemia with aortic stiffness remains to be further clarified, it seems to be triggered by the elevated oxidation and inflammation levels of vascular endothelial cells, which lack in nitric oxide production and availability (194).

Increased risk of vein thrombosis has been also connected with hyperhomocysteinemia. It has been indicated that elevated levels of homocysteine can enhance platelet adhesion on endothelial cells, while promoting the production of prothrombotic factors, such as tissue plasminogen activator and β-thromboglobulin (194, 195).

### MicroRNAs

MicroRNAs are small non-coding molecules involved in the regulation of numerous genes due to their ability to recognize target sequences situated within the 3 prime untranslated region (3′-UTR) of messenger RNA (mRNA). miRNAs have a regulating effect in the post-transcriptional expression of eukaryotic genes and their role in PCOS patients is prominent. In a recent study performed in 2015, where 25 women with PCOS were compared with 24 healthy women of the same age and weight, an increased presence of miRNA-93 and miRNA-223 has been observed in the group of women with PCOS. The p values for these observations were <0.01 and 0.029 respectively, indicating miRNA-93 as a better circulating biomarker for the detection of PCOS (196). The upregulation of miRNA-93 induces insulin resistance, through targeting the CDKN1A and GLUT4 genes, therefore contributing to the increased risk of CVD in PCOS patients (197).

On the other hand, miRNA-223 which targets glucose transporter type 4 (GLUT4), has also been found significantly upregulated in patients with type II diabetes mellitus (T2DM) and left ventricular heart dysfunction (LVD) in biopsies from the left ventricle. When the effect of miRNA-223 was studied *in vivo* in rat cardiomyocytes, a GLUT4 mediated glucose uptake increase has been found as a response to miRNA-223 upregulation. The regulatory function of miRNA-223 on the post-transcriptional expression of GLUT4 and subsequently on glucose uptake was validated using a synthetic inhibitor of the miRNA *in vivo*, which diminished the levels of GLUT4 and glucose uptake (198).

Apart from miRNA-93 and miRNA-223, several other miRNAs have been found to be differentially expressed in women with PCOS in the follicular fluid. The most significant ones, which have been observed to demonstrate a more than 2-fold change, are miRNA-199b, miRNA-650, miRNA-663b, miRNA-361, miRNA-127, miRNA-382, miRNA-425, miRNA-212, miRNA-891b, miRNA-513c, miRNA-507, miRNA-32, miRNA-200c (199).

In a recent meta-analysis performed in 2020, two new miRNAs have been proposed as potential diagnostic biomarkers for PCOS, miR-29a-5p, and miR-320, respectively, indicating miR-29a-5p as a superior potential biomarker (200). Both molecules seem to be downregulated in patients with PCOS. There is a connection between miR-320 and the regulation of genes associated with PCOS morbidity, whereas miR-29a-5p is involved in several metabolic diseases and comorbidities. Moreover, the significant role of miR-29a-5p regarding cell growth, differentiation, and proliferation has also been highlighted. When the DIANA-microT-CDS tool was used for the determination of differentially expressed target genes, which are involved in pathways targeted by miRNAs and associated with PCOS, several results came up. In particular, miR-320 was found to possibly interact with the expression of ESR1, IL-1A, 10, 12B, 37, 8, RAB5B, PDK3, and HMGA2, all of which are involved in estradiol synthesis, steroidogenesis, insulin signaling, fertilization, cell adhesion, and embryo development. On the other hand, miR-29a-5p was found to potentially regulate AR, AKT2, TGFβ, MAP, KFBN3, STARD3, ITGB1, TGFB2, and INRS, which are involved in follicle growth, cell growth, insulin, and collagen synthesis (200).

Nowadays, the correlation between miRNAs and different pathological conditions has been profoundly studied, connecting the dysregulated expression of miRNAs with complex diseases, including CVD. Usually, the targets of a miRNA expand on several different mRNAs, thus affecting the expression of a collection of genes. It has been estimated that around 30% of genes are regulated by miRNAs (201), depicting their significance in human physiology. A promising field for future research is the monitoring of serum miRNAs, so as to be used as diagnostic, prognostic, or treatment response markers.

## Conclusion

Polycystic ovary syndrome is one of the most common endocrine disorders in women of childbearing age and the most common source of anovulatory infertility. This syndrome presents heterogeneous clinical variants, where the pathogenesis involves the existence of several cardiometabolic abnormalities that manifest through changes in the metabolic balance in which mitochondrial dysfunctions play a key role in the progression and occurrence of complications. Besides mitochondrial dysfunction, systemic inflammation characteristic of PCOS women also fulfills an important role in the occurrence of complications associated with metabolic disorders in these patients, as well as in the predisposition to cardiovascular disease.

Among the metabolic disorders associated with PCOS that occur from adolescence, insulin resistance and impaired glucose tolerance are included, as well as other manifestations that are more prominently expressed with age, such as hyperglycemia, obesity - especially visceral, hepatic steatosis, dyslipidemia, hypertension, type II diabetes, and an increased risk of cardiovascular diseases such as hypertension and myocardial infarction. Moreover, in addition to other features such as chronic low-grade inflammatory state and endothelial dysfunction, PCOS poses an increased risk of developing cardiovascular disorders. One of the diverse mechanisms that could enhance the overall cardiovascular risk especially by causing arterial hypertension is represented by endothelial dysfunction, which is tightly correlated with ROS levels that are highly dependent upon the oxidative stress in the body. In this respect, high ROS levels are further involved in genetic changes such as point mutations, DNA strand breaks, aberrant DNA cross-linking, and DNA-protein cross-linking, DNA methylation, with the effect of silencing the genes tumor suppressors, phenomena that were observed in women with PCOS syndrome. Moreover, owing to OS’s ability to induce DNA injury and methylation, the activation of oncogenes along with antioncogene silencing are not out of the question among these patients, which are, in fact, also susceptible to developing endometrial cancer.

Overall, current literature suggests an evident increase in OS among PCOS women, contributing to the numerous metabolic and cardiovascular dysfunctions characteristic of this disease. The development of both preventive and therapeutic strategies aimed at the cardiovascular risk of these patients ought to therefore involve further studies regarding the reduction of oxidative stress.

## Author Contributions

Conceptualization, FD, AB, CD, PA. Methodology, SC, VV. Investigation, SO, DC. Writing—original draft preparation, FD, CD, AB, PA, CC. Writing—review and editing CC, SO. Supervision, DC. Funding acquisition, NS, SC. All authors contributed to the article and approved the submitted version.

## Funding

This work was supported by grants of the Romanian Ministry of Research and Innovation, CCCDI-UEFISCDI, project number PN-III-P1-1.2-PCCDI-2017-0833/68/2018.

## Conflict of Interest

PA was employed by the company Antisel RO SRL, Division of Molecular Diagnostics and Biotechnology, Bucharest, Romania.

The remaining authors declare that the research was conducted in the absence of any commercial or financial relationships that could be construed as a potential conflict of interest.

## References

[1] Senoner T Dichtl W. Oxidative Stress in Cardiovascular Diseases: Still a Therapeutic Target? Nutrients (2019) 11:2090. 10.3390/nu11092090 PMC6769522 31487802

[2] Finkel T. Signal transduction by reactive oxygen species. J Cell Biol (2011) 194:7–15. 10.1083/jcb.201102095 21746850 PMC3135394

[3] Tsutsui H Kinugawa S Matsushima S. Oxidative stress and heart failure. Am J Physiol Heart Circ Physiol (2011) 301:H2181–90. 10.1152/ajpheart.00554.2011 21949114

[4] Samman Tahhan A Sandesara PB Hayek SS Alkhoder A Chivukula K Hammadah M. Association between oxidative stress and atrial fibrillation. Heart Rhythm (2017) 14:1849–55. 10.1016/j.hrthm.2017.07.028 PMC5817893 28757307

[5] Baradaran A Nasri H Rafieian-Kopaei M. Oxidative stress and hypertension: Possibility of hypertension therapy with antioxidants. J Res Med Sci (2014) 19:358–67. PMC4115353 25097610

[6] Kattoor AJ Pothineni NVK Palagiri D Mehta JL. Oxidative Stress in Atherosclerosis. Curr Atheroscl Rep (2017) 19:42. 10.1007/s11883-017-0678-6 28921056

[7] Ashok V Ranganathan R Chander S Damodar S Bhat S Nataraj KS,. Comparison of Diagnostic Yield of a FISH Panel Against Conventional Cytogenetic Studies for Hematological Malignancies: A South Indian Referral Laboratory Analysis Of 201 Cases. Asian Pacific J Cancer Prev APJCP (2017) 18:3457–64. 10.22034/APJCP.2017.18.12.3457 PMC5980910 29286619

[8] Godo S Shimokawa H. Endothelial Functions. Arterioscler Thromb Vasc Biol (2017) 37:e108–14. 10.1161/atvbaha.117.309813 28835487

[9] Çetin M Tunçdemir P Karaman K Yel S Karaman E Özgökçe M. Cardiovascular evaluation and serum paraoxonase-1 levels in adolescents with polycystic ovary syndrome. J Obstetr Gynaecol (2020) 40:90–5. 10.1080/01443615.2019.1604643 31215308

[10] Osibogun O Ogunmoroti O Michos E. Polycystic Ovary Syndrome and Cardiometabolic Risk: Opportunities for Cardiovascular Disease Prevention. Trends Cardiovasc Med (2019) 30(7):399–404. 10.1016/j.tcm.2019.08.010 31519403

[11] McCartney CR Campbell RE. Abnormal GnRH Pulsatility in Polycystic Ovary Syndrome: Recent Insights. Curr Opin Endocr Metab Res (2020) 12:78–84. 10.1016/j.coemr.2020.04.005 32676541 PMC7365617

[12] Ho CH Chang CM Li HY Shen HY Lieu FK Wang PS. Dysregulated immunological and metabolic functions discovered by a polygenic integrative analysis for PCOS. Reprod Biomed Online (2020) 40:160–7. 10.1016/j.rbmo.2019.09.011 31780352

[13] Zhang J Bao Y Zhou X Zheng L. Polycystic ovary syndrome and mitochondrial dysfunction. Reprod Biol Endocrinol (2019) 17:67. 10.1186/s12958-019-0509-4 31420039 PMC6698037

[14] Yildiz BO. Diagnosis of hyperandrogenism: clinical criteria. Best Pract Res Clin Endocrinol Metab (2006) 20:167–76. 10.1016/j.beem.2006.02.004 16772149

[15] Escobar-Morreale HF San Millán JL. Abdominal adiposity and the polycystic ovary syndrome. Trends Endocrinol Metabol: TEM (2007) 18:266–72. 10.1016/j.tem.2007.07.003 17693095

[16] Vázquez-Vela ME Torres N Tovar AR. White adipose tissue as endocrine organ and its role in obesity. Arch Med Res (2008) 39:715–28. 10.1016/j.arcmed.2008.09.005 18996284

[17] Spritzer P Lecke S Satler F Morsch D. Adipose tissue dysfunction, adipokines and low-grade chronic inflammation in PCOS. Reprod (Cambridge England) (2015) 149(5):R219–27. 10.1530/REP-14-0435 25628442

[18] Ziech D Franco R Pappa A Panayiotidis MI. Reactive oxygen species (ROS)–induced genetic and epigenetic alterations in human carcinogenesis. Mutat Res (2011) 711:167–73. 10.1016/j.mrfmmm.2011.02.015 21419141

[19] Lebedeva MA Eaton JS Shadel GS. Loss of p53 causes mitochondrial DNA depletion and altered mitochondrial reactive oxygen species homeostasis. Biochim Biophys Acta (2009) 1787:328–34. 10.1016/j.bbabio.2009.01.004 PMC2680458 19413947

[20] Donkena KV Young CY Tindall DJ. Oxidative stress and DNA methylation in prostate cancer. Obstetr Gynecol Int (2010) 2010:302051. 10.1155/2010/302051 PMC2910495 20671914

[21] Franco R Schoneveld O Georgakilas AG Panayiotidis MI. Oxidative stress, DNA methylation and carcinogenesis. Cancer Lett (2008) 266:6–11. 10.1016/j.canlet.2008.02.026 18372104

[22] Bartsch H Nair J. Chronic inflammation and oxidative stress in the genesis and perpetuation of cancer: Role of lipid peroxidation, DNA damage, and repair. Langenbeck’s Arch Surg Deutsche Gesellschaft Für Chirurgie (2006) 391:499–510. 10.1007/s00423-006-0073-1 16909291

[23] Goodarzi MO Dumesic DA Chazenbalk G Azziz R. Polycystic ovary syndrome: etiology, pathogenesis and diagnosis. Nat Rev Endocrinol (2011) 7:219–31. 10.1038/nrendo.2010.217 21263450

[24] McCartney CR Marshall JC. CLINICAL PRACTICE. Polycystic Ovary Syndrome. N Engl J Med (2016) 375:54–64. 10.1056/NEJMcp1514916 27406348 PMC5301909

[25] Barthelmess EK Naz RK. Polycystic ovary syndrome: current status and future perspective. Front Biosci (Elite Ed) (2014) 6:104–19. 10.2741/e695 PMC4341818 24389146

[26] Witchel SF Oberfield S Rosenfield RL Codner E Bonny A Ibáñez L. The Diagnosis of Polycystic Ovary Syndrome during Adolescence. Hormone Res Paediatr (2015) 83:376–89. 10.1159/000375530 25833060

[27] Strauss JF 3rd. Some new thoughts on the pathophysiology and genetics of polycystic ovary syndrome. Ann New Y Acad Sci (2003) 997:42–8. 10.1196/annals.1290.005 14644808

[28] Witchel SF Oberfield SE Peña AS. Polycystic Ovary Syndrome: Pathophysiology, Presentation, and Treatment With Emphasis on Adolescent Girls. J Endocr Soc (2019) 3:1545–73. 10.1210/js.2019-00078 PMC6676075 31384717

[29] Bednarska S Siejka A. The pathogenesis and treatment of polycystic ovary syndrome: What’s new? Adv Clin Exp Med (2017) 26:359–67. 10.17219/acem/59380 28791858

[30] Mihailidis J Dermesropian R Taxel P Luthra P Grant-Kels JM. Endocrine evaluation of hirsutism. Int J Women’s Dermatol (2017) 3:S6–S10. 10.1016/j.ijwd.2017.02.007 28492032 PMC5419053

[31] Binita G Suprava P Mainak C Koner BC Alpana S. Correlation of prolactin and thyroid hormone concentration with menstrual patterns in infertile women. J Reprod Infertil (2009) 10:207–12. 10.18203/2320-1770.ijrcog20170400 PMC3719326 23926470

[32] Dumesic DA Oberfield SE Stener-Victorin E Marshall JC Laven JS Legro RS. Scientific Statement on the Diagnostic Criteria, Epidemiology, Pathophysiology, and Molecular Genetics of Polycystic Ovary Syndrome. Endocrine Rev (2015) 36:487–525. 10.1210/er.2015-1018 26426951 PMC4591526

[33] Rojas J Chávez M Olivar L Rojas M Morillo J Mejías J. Polycystic Ovary Syndrome, Insulin Resistance, and Obesity: Navigating the Pathophysiologic Labyrinth. Int J Reprod Med (2014) 2014:719050. 10.1155/2014/719050 25763405 PMC4334071

[34] Shaaban Z Khoradmehr A Jafarzadeh Shirazi MR Tamadon A. Pathophysiological mechanisms of gonadotropins- and steroid hormones-related genes in etiology of polycystic ovary syndrome. Iran J Basic Med Sci (2019) 22:3–16. 10.22038/ijbms.2018.31776.7646 30944702 PMC6437453

[35] Fenichel P Rougier C Hieronimus S Chevalier N. Which origin for polycystic ovaries syndrome: Genetic, environmental or both? Annales D’endocrinol (2017) 78:176–85. 10.1016/j.ando.2017.04.024 28606381

[36] Hoffman B Schorge J Schaffer J Halvorson L Bradshaw K Cunningham F. Williams Gynecology. 2nd ed. New York: Mcgraw-hill (2012).

[37] Cheung AP Cog F. Polycystic ovary syndrome: a contemporary view. J Obstetr Gynaecol Canada JOGC J D’obstetr Gynecol Du Canada JOGC (2010) 32:423–5. 10.1016/s1701-2163(16)34493-0 20500948

[38] Zheng L Annab LA Afshari CA Lee WH Boyer TG. BRCA1 mediates ligand-independent transcriptional repression of the estrogen receptor. Proc Natl Acad Sci USA (2001) 98:9587–92. 10.1073/pnas.171174298 PMC55496 11493692

[39] Chung TK Lau TS Cheung TH Yim SF Lo KW Siu NS. Dysregulation of microRNA-204 mediates migration and invasion of endometrial cancer by regulating FOXC1. Int J Cancer (2012) 130:1036–45. 10.1002/ijc.26060 21400511

[40] Moore AM Campbell RE. The neuroendocrine genesis of polycystic ovary syndrome: A role for arcuate nucleus GABA neurons. J Steroid Biochem Mol Biol (2016) 160:106–17. 10.1016/j.jsbmb.2015.10.002 26455490

[41] Sliwowska JH Fergani C Gawałek M Skowronska B Fichna P Lehman MN. Insulin: its role in the central control of reproduction. Physiol Behav (2014) 133:197–206. 10.1016/j.physbeh.2014.05.021 24874777 PMC4084551

[42] Roland AV Moenter SM. Reproductive neuroendocrine dysfunction in polycystic ovary syndrome: insight from animal models. Front Neuroendocrinol (2014) 35:494–511. 10.1016/j.yfrne.2014.04.002 24747343 PMC4175187

[43] Ubuka T Morgan K Pawson AJ Osugi T Chowdhury VS Minakata H. Identification of human GnIH homologs, RFRP-1 and RFRP-3, and the cognate receptor, GPR147 in the human hypothalamic pituitary axis. PLoS One (2009) 4:e8400. 10.1371/journal.pone.0008400 20027225 PMC2791420

[44] Catteau-Jonard S Dewailly D. Pathophysiology of Polycystic Ovary Syndrome: The Role of Hyperandrogenism. Front Horm Res (2013) 40:22–7. 10.1159/000341679 24002402

[45] Mehrabian F Afghahi M. Can Sex-hormone Binding Globulin Considered as a Predictor of Response to Pharmacological Treatment in Women with Polycystic Ovary Syndrome? Int J Prev Med (2013) 4:1169–74. PMC3843304 24319557

[46] Lerchbaum E Schwetz V Rabe T Giuliani A Obermayer-Pietsch B. Hyperandrogenemia in polycystic ovary syndrome: exploration of the role of free testosterone and androstenedione in metabolic phenotype. PLoS One (2014) 9:e108263–e108263. 10.1371/journal.pone.0108263 25310562 PMC4195601

[47] Filippou P Homburg R. Is foetal hyperexposure to androgens a cause of PCOS? Hum Reprod Update (2017) 23:421–32. 10.1093/humupd/dmx013 28531286

[48] Howland MA Sandman CA Glynn LM. Developmental origins of the human hypothalamic-pituitary-adrenal axis. Expert Rev Endocrinol Metab (2017) 12:321–39. 10.1080/17446651.2017.1356222 PMC6334849 30058893

[49] Puttabyatappa M Cardoso RC Padmanabhan V. Effect of maternal PCOS and PCOS-like phenotype on the offspring’s health. Mol Cell Endocrinol (2016) 435:29–39. 10.1016/j.mce.2015.11.030 26639019 PMC4884168

[50] Gourgari E Lodish M Keil M Sinaii N Turkbey E Lyssikatos C. Bilateral Adrenal Hyperplasia as a Possible Mechanism for Hyperandrogenism in Women With Polycystic Ovary Syndrome. J Clin Endocrinol Metab (2016) 101:3353–60. 10.1210/jc.2015-4019 PMC5010568 27336356

[51] De Leo V Musacchio MC Cappelli V Massaro MG Morgante G Petraglia F. Genetic, hormonal and metabolic aspects of PCOS: an update. Reprod Biol Endocrinol (2016) 14:38–8. 10.1186/s12958-016-0173-x PMC4947298 27423183

[52] Rosenfield RL Ehrmann DA. The Pathogenesis of Polycystic Ovary Syndrome (PCOS): The Hypothesis of PCOS as Functional Ovarian Hyperandrogenism Revisited. Endocrine Rev (2016) 37:467–520. 10.1210/er.2015-1104 27459230 PMC5045492

[53] Deligeoroglou E Kouskouti C Christopoulos P. The role of genes in the polycystic ovary syndrome: predisposition and mechanisms. Gynecolog Endocrinol (2009) 25:603–9. 10.1080/09513590903015619 19591017

[54] Stocco C. Tissue physiology and pathology of aromatase. Steroids (2012) 77:27–35. 10.1016/j.steroids.2011.10.013 22108547 PMC3286233

[55] Spritzer PM Lecke SB Satler F Morsch DM. Adipose tissue dysfunction, adipokines, and low-grade chronic inflammation in polycystic ovary syndrome. Reproduction (2015) 149:R219. 10.1530/rep-14-0435 25628442

[56] Wilcox G. Insulin and insulin resistance. Clin Biochem Rev (2005) 26:19–39. 16278749 PMC1204764

[57] Burcelin R Thorens B Glauser M Gaillard R Pralong F. Gonadotropin-Releasing Hormone Secretion from Hypothalamic Neurons: Stimulation by Insulin and Potentiation by Leptin. Endocrinology (2003) 144:4484–91. 10.1210/en.2003-0457 12960084

[58] Marshall JC Dunaif A. Should all women with PCOS be treated for insulin resistance? Fertil Steril (2012) 97:18–22. 10.1016/j.fertnstert.2011.11.036 22192137 PMC3277302

[59] Toprak S Yönem A Cakir B Güler S Azal O Ozata M. Insulin resistance in nonobese patients with polycystic ovary syndrome. Hormone Res (2001) 55:65–70. 10.1159/000049972 11509861

[60] Ashraf S Nabi M Rasool S Rashid F Amin,S. Hyperandrogenism in polycystic ovarian syndrome and role of CYP gene variants: a review. Egyptian J Med Hum Genet (2019) 20:25. 10.1186/s43042-019-0031-4

[61] Srimyooran Branavan U Nv C Wss W Wijeyaratne C. Polycystic Ovary Syndrome: Genetic Contributions from the Hypothalamic-Pituitary-Gonadal Axis. Int Arch Endocrinol Clin Res (2018) 4:013. 10.23937/2572-407X.1510013

[62] Ruddenklau A Campbell RE. Neuroendocrine Impairments of Polycystic Ovary Syndrome. Endocrinology (2019) 160:2230–42. 10.1210/en.2019-00428 31265059

[63] Cornier M-A Dabelea D Hernandez TL Lindstrom RC Steig AJ Stob NR. The metabolic syndrome. Endocrine Rev (2008) 29:777–822. 10.1210/er.2008-0024 18971485 PMC5393149

[64] Geffner ME Golde DW. Selective insulin action on skin, ovary, and heart in insulin-resistant states. Diabetes Care (1988) 11:500–5. 10.2337/diacare.11.6.500 2969796

[65] Wu S Divall S Wondisford F Wolfe A. Reproductive Tissues Maintain Insulin Sensitivity in Diet-Induced Obesity. Diabetes (2012) 61:114–23. 10.2337/db11-0956 PMC3237653 22076926

[66] Richards JS Ren YA Candelaria N Adams JE Rajkovic A. Ovarian Follicular Theca Cell Recruitment, Differentiation, and Impact on Fertility: 2017 Update. Endocrine Rev (2018) 39:1–20. 10.1210/er.2017-00164 29028960 PMC5807095

[67] Lindheim SR Glenn TL Smith MC Gagneux P. Ovulation Induction for the General Gynecologist. J Obstet Gynaecol India (2018) 68:242–52. 10.1007/s13224-018-1130-8 PMC6046677 30065537

[68] Franks S Stark J Hardy K. Follicle dynamics and anovulation in polycystic ovary syndrome. Hum Reprod Update (2008) 14:367–78. 10.1093/humupd/dmn015 18499708

[69] Park JC Lim SY Jang TK Bae JG Kim JI Rhee JH. Endometrial histology and predictable clinical factors for endometrial disease in women with polycystic ovary syndrome. Clin Exp Reprod Med (2011) 38:42–6. 10.5653/cerm.2011.38.1.42 PMC3283042 22384417

[70] Monniaux D Cadoret V Clément F Dalbies-Tran R Elis S Fabre S. “Folliculogenesis”. In: Huhtaniemi I Martini L, editors. Encyclopedia of Endocrine Diseases, 2nd ed. Oxford: Academic Press (2019). p. 377–98. 10.1016/B978-0-12-801238-3.64550-6pp

[71] Kumar P Sait SF. Luteinizing hormone and its dilemma in ovulation induction. J Hum Reprod Sci (2011) 4:2–7. 10.4103/0974-1208.82351 21772731 PMC3136063

[72] Lopez H Sartori R Wiltbank MC. Reproductive Hormones and Follicular Growth During Development of One or Multiple Dominant Follicles in Cattle1. Biol Reprod (2005) 72:788–95. 10.1095/biolreprod.104.035493 15525815

[73] Jonard S Dewailly D. The follicular excess in polycystic ovaries, due to intra-ovarian hyperandrogenism, may be the main culprit for the follicular arrest. Hum Reprod Update (2004) 10:107–17. 10.1093/humupd/dmh010 15073141

[74] Dumesic DA Lesnick TG Stassart JP Ball GD Wong A Abbott DH. Intrafollicular antimüllerian hormone levels predict follicle responsiveness to follicle-stimulating hormone (FSH) in normoandrogenic ovulatory women undergoing gonadotropin releasing-hormone analog/recombinant human FSH therapy for in vitro fertilization and embryo transfer. Fertil Steril (2009) 92:217–21. 10.1016/j.fertnstert.2008.04.047 PMC2703699 18675414

[75] Diamanti-Kandarakis E. Polycystic ovarian syndrome: pathophysiology, molecular aspects and clinical implications. Expert Rev Mol Med (2008) 10:e3. 10.1017/s1462399408000598 18230193

[76] Dupont J Scaramuzzi RJ. Insulin signalling and glucose transport in the ovary and ovarian function during the ovarian cycle. Biochem J (2016) 473:1483–501. 10.1042/BCJ20160124 PMC4888492 27234585

[77] Welt CK Taylor AE Fox J Messerlian GM Adams JM Schneyer AL. Follicular Arrest in Polycystic Ovary Syndrome Is Associated with Deficient Inhibin A and B Biosynthesis. J Clin Endocrinol Metab (2005) 90:5582–7. 10.1210/jc.2005-0695 16030174

[78] Webber LJ Stubbs S Stark J Trew GH Margara R Hardy K. Formation and early development of follicles in the polycystic ovary. Lancet (London England) (2003) 362:1017–21. 10.1016/s0140-6736(03)14410-8 14522531

[79] Jonard S Dewailly D. The follicular excess in polycystic ovaries, due to intra-ovarian hyperandrogenism, may be the main culprit for the follicular arrest. Hum Reprod Update (2004) 10:107–17. 10.1093/humupd/dmh010 15073141

[80] Johansson J Stener-Victorin E. Polycystic ovary syndrome: effect and mechanisms of acupuncture for ovulation induction. Evid Based Complement Alternat Med (2013) 2013:762615–5. 10.1155/2013/762615 PMC3773899 24073009

[81] Tsutsumi R Webster NJG. GnRH pulsatility, the pituitary response and reproductive dysfunction. Endocr J (2009) 56:729–37. 10.1507/endocrj.k09e-185 PMC4307809 19609045

[82] Lashen H. Role of metformin in the management of polycystic ovary syndrome. Ther Adv Endocrinol Metab (2010) 1:117–28. 10.1177/2042018810380215 PMC3475283 23148156

[83] Johnson NP. Metformin use in women with polycystic ovary syndrome. Ann Trans Med (2014) 2:56–6. 10.3978/j.issn.2305-5839.2014.04.15 PMC4200666 25333031

[84] Duleba AJ Dokras A. Is PCOS an inflammatory process? Fertil Steril (2012) 97:7–12. 10.1016/j.fertnstert.2011.11.023 22192135 PMC3245829

[85] Wild RA Carmina E Diamanti-Kandarakis E Dokras A Escobar-Morreale HF Futterweit W. Assessment of cardiovascular risk and prevention of cardiovascular disease in women with the polycystic ovary syndrome: a consensus statement by the Androgen Excess and Polycystic Ovary Syndrome (AE-PCOS) Society. J Clin Endocrinol Metab (2010) 95:2038–49. 10.1210/jc.2009-2724 20375205

[86] Hulsmans M Holvoet P. The vicious circle between oxidative stress and inflammation in atherosclerosis. J Cell Mol Med (2010) 14:70–8. 10.1111/j.1582-4934.2009.00978.x PMC3837590 19968738

[87] Garcia M Mulvagh SL Merz CNB Buring JE Manson JE. Cardiovascular Disease in Women: Clinical Perspectives. Circ Res (2016) 118:1273–93. 10.1161/CIRCRESAHA.116.307547 PMC4834856 27081110

[88] Pathak E. Is Heart Disease or Cancer the Leading Cause of Death in United States Women? Women Health Issues (2016) 26(6):589–94. 10.1016/j.whi.2016.08.002 27717539

[89] Abuful A Gidron Y Henkin Y. Physicians’ attitudes toward preventive therapy for coronary artery disease: is there a gender bias? Clin Cardiol (2005) 28:389–93. 10.1002/clc.4960280809 PMC6654010 16144216

[90] Chou AF Scholle SH Weisman CS Bierman AS Correa-de-Araujo R Mosca L. Gender disparities in the quality of cardiovascular disease care in private managed care plans. Women’s Health Issues Off Publ Jacobs Institute Women’s Health (2007) 17:120–30. 10.1016/j.whi.2007.03.002 17448685

[91] Gu Q Burt VL Paulose-Ram R Dillon CF. Gender differences in hypertension treatment, drug utilization patterns, and blood pressure control among US adults with hypertension: data from the National Health and Nutrition Examination Survey 1999-2004. Am J Hypertension (2008) 21:789–98. 10.1038/ajh.2008.185 18451806

[92] Mosca L Benjamin EJ Berra K Bezanson JL Dolor RJ Lloyd-Jones DM. Effectiveness-based guidelines for the prevention of cardiovascular disease in women–2011 update: a guideline from the american heart association. Circulation (2011) 123:1243–62. 10.1161/CIR.0b013e31820faaf8 PMC3182143 21325087

[93] Keteepe-Arachi T Sharma S. Cardiovascular Disease in Women: Understanding Symptoms and Risk Factors. Eur Cardiol (2017) 12:10–3. 10.15420/ecr.2016:32:1 PMC6206467 30416543

[94] Pinola P Puukka K Piltonen TT Puurunen J Vanky E Sundström-Poromaa I. Normo- and hyperandrogenic women with polycystic ovary syndrome exhibit an adverse metabolic profile through life. Fertil Steril (2017) 107(3):788–95.e782. 10.1016/j.fertnstert.2016.12.017 28089571

[95] Schmidt J Landin-Wilhelmsen K Brannstrom M Dahlgren E. Cardiovascular disease and risk factors in PCOS women of postmenopausal age: a 21-year controlled follow-up study. J Clin Endocrinol Metab (2011) 96(12):3794–803. 10.1210/jc.2011-1677 21956415

[96] Brown ZA Louwers YV Fong SL Valkenburg O Birnie E de Jong FH. The phenotype of polycystic ovary syndrome ameliorates with aging. Fertil Steril (2011) 96(5):1259–65. 10.1016/j.fertnstert.2011.09.002 21963227

[97] Meun C Franco OH Dhana K Jaspers L Muka T Louwers Y. High Androgens in Postmenopausal Women and the Risk for Atherosclerosis and Cardiovascular Disease: The Rotterdam Study. J Clin Endocrinol Metab (2018) 103(4):1622–30. 10.1210/jc.2017-02421 29408955

[98] Zhao Y Qiao J. Ethnic differences in the phenotypic expression of polycystic ovary syndrome. Steroids (2013) 78(8):755–60. 10.1016/j.steroids.2013.04.006 23624030

[99] Fauser BC Tarlatzis BC Rebar RW Legro RS Balen AH Lobo R. Consensus on women’s health aspects of polycystic ovary syndrome (PCOS): the Amsterdam ESHRE/ASRM-Sponsored 3rd PCOS Consensus Workshop. Group Fertil Steril (2012) 97(1):28–38.e25. 10.1016/j.fertnstert.2011.09.024 22153789

[100] Wild RA. Polycystic ovary syndrome: a risk for coronary artery disease? Am J Obstetr Gynecol (2002) 186:35–43. 10.1067/mob.2002.119180 11810081

[101] Chiu WL Boyle J Vincent A Teede H Moran LJ. Cardiometabolic Risks in Polycystic Ovary Syndrome: Non-Traditional Risk Factors and the Impact of Obesity. Neuroendocrinology (2017) 104:412–24. 10.1159/000455233 28006770

[102] Palomba S Santagni S Falbo A La Sala GB. Complications and challenges associated with polycystic ovary syndrome: current perspectives. Int J Womens Health (2015) 7:745–63. 10.2147/IJWH.S70314 PMC4527566 26261426

[103] Fauser BC Tarlatzis BC Rebar RW Legro RS Balen AH Lobo R. Consensus on women’s health aspects of polycystic ovary syndrome (PCOS): the Amsterdam ESHRE/ASRM-Sponsored 3rd PCOS Consensus Workshop Group. Fertil Steril (2012) 97:28–38.e25. 10.1016/j.fertnstert.2011.09.024 22153789

[104] Bentley-Lewis R Seely E Dunaif A. Ovarian hypertension: polycystic ovary syndrome. Endocrinol Metab Clin North Am (2011) 40(2):433–x. 10.1016/j.ecl.2011.01.009 PMC3253555 21565677

[105] Orbetzova MM Shigarminova RG Genchev GG Milcheva BA Lozanov LB Genov NS. Role of 24-hour monitoring in assessing blood pressure changes in polycystic ovary syndrome. Folia Med (Plovdiv) (2003) 45(3):21–5. 15366662

[106] Wild S Pierpoint T Jacobs H McKeigue P. Long-term consequences of polycystic ovary syndrome: results of a 31 year follow-up study. Hum Fertil (Camb) (2000) 3(2):101–5. 10.1080/1464727002000198781 11844363

[107] Holte J Gennarelli G Berne C Bergh T Lithell H. Elevated ambulatory day-time blood pressure in women with polycystic ovary syndrome: a sign of a pre-hypertensive state? Hum Reprod (1996) 11(1):23–8. 10.1093/oxfordjournals.humrep.a019028 8671152

[108] Elting MW Korsen TJ Bezemer PD Schoemaker J. Prevalence of diabetes mellitus, hypertension and cardiac complaints in a follow-up study of a Dutch PCOS population. Hum Reprod (2001) 16(3):556–60. 10.1093/humrep/16.3.556 11228228

[109] Vrbíková J Cífková R Jirkovská A Lánská V Platilová H Zamrazil V. Cardiovascular risk factors in young Czech females with polycystic ovary syndrome. Hum Reprod (2003) 18(5):980–4. 10.1093/humrep/deg218 12721172

[110] Talbott E Clerici A Berga SL Kuller L Guzick D Detre K. Adverse lipid and coronary heart disease risk profiles in young women with polycystic ovary syndrome: results of a case-control study. J Clin Epidemiol (1998) 51(5):415–22. 10.1016/S0895-4356(98)00010-9 9619969

[111] Conway GS Agrawal R Betteridge DJ Jacobs HS. Risk factors for coronary artery disease in lean and obese women with the polycystic ovary syndrome. ClinEndocrinol (Oxf) (1992) 37(2):119–25. 10.1111/j.1365-2265.1992.tb02295.x 1395062

[112] Amiri M Ramezani Tehrani F Behboudi-Gandevani S Bidhendi-Yarandi R Carmina E. Risk of hypertension in women with polycystic ovary syndrome: a systematic review, meta-analysis and meta-regression. Reprod Biol Endocrinol (2020) 18(1):23. 10.1186/s12958-020-00576-1 32183820 PMC7076940

[113] Sam S. Obesity and Polycystic Ovary Syndrome. Obes Manag (2007) 3(2):69–73. 10.1089/obe.2007.0019 20436797 PMC2861983

[114] Lim SS Davies MJ Norman RJ Moran LJ. Overweight, obesity and central obesity in women with polycystic ovary syndrome: a systematic review and meta–analysis. Hum Reprod Update (2012) 18(6):618–37. 10.1093/humupd/dms030 22767467

[115] Rachon D Teede H. Ovarian function and obesity–interrelationship, impact on women’s reproductive lifespan and treatment options. Molec Cell Endocrinol (2010) 316(2):172–9. 10.1016/j.mce.2009.09.026 19818376

[116] Yildirir A Aybar F Kabakci G Yarali H Oto A. Heart rate variability in young women with polycystic ovary syndrome. Ann Noninvasive Electrocardiol (2006) 11(4):306–12. 10.1111/j.1542-474X.2006.00122.x PMC6932634 17040278

[117] Muller-Wieland D Kotzka J Knebel B Krone W. Metabolic syndrome and hypertension: pathophysiology and molecular basis of insulin resistance. Basic Res Cardiol (1998) 93(Suppl 2):131–4. 10.1007/s003950050238 9833175

[118] Muniyappa R Montagnani M Koh KK Quon MJ. Cardiovascular actions of insulin. Endocr Rev (2007) 28(5):463–91. 10.1210/er.2007-0006 17525361

[119] Barnes RB Namnoum AB Rosenfield RL Layman LC. The role of LH and FSH in ovarian androgen secretion and ovarian follicular development: Clinical studies in a patient with isolated FSH deficiency and multicystic ovaries: Case report. Hum Reprod (2002) 17:88–91. 10.1093/humrep/17.1.88 11756367

[120] Burt Solorzano CM McCartney CR Blank SK Knudsen KL Marshall JC. Hyperandrogenaemia in adolescent girls: origins of abnormal gonadotropin-releasing hormone secretion. BJOG Int J Obstetr Gynaecol (2010) 117:143–9. 10.1111/j.1471-0528.2009.02383.x PMC2994606 20002394

[121] Jaliseh HK Tehrani FR Behboudi-Gandevani S Hosseinpanah F Khalili D Cheraghi L. Polycystic ovary syndrome is a risk factor for diabetes and prediabetes in middle-aged but not elderly women: a long-term population-based follow-up study. Fertil Steril (2017) 108(6):1078–84. 10.1016/j.fertnstert.2017.09.004 29202960

[122] Joham AE Boyle JA Zoungas S Teede HJ. Hypertension in reproductive aged women with polycystic ovary syndrome and association with obesity. Am J Hypertens (2014) 28(7):847–51. 10.1093/ajh/hpu251 25542625

[123] Winters SJ Talbott E Guzick DS Zborowski J McHugh KP. Serum testosterone levels decrease in middle age in women with the polycystic ovary syndrome. Fertil Steril (2000) 73:724–9. 10.1016/s0015-0282(99)00641-x 10731532

[124] Carmina E. Cardiovascular risk and events in polycystic ovary syndrome. Climacteric (2009) 12(sup1):22–5. 10.1080/13697130903003842 19811236

[125] Carmina E Campagna A Lobo R. Emergence of ovulatory cycles with aging in women with polycystic ovary syndrome (PCOS) alters the trajectory of cardiovascular and metabolic risk factors. Hum Reprod (2013) 28(8):2245–52. 10.1093/humrep/det119 23595974

[126] Lo JC Feigenbaum SL Yang J Pressman AR Selb JV Go AS. Epidemiology and adverse cardiovascular risk profile of diagnosed polycystic ovary syndrome. J Clin Endocrinol Metab (2006) 91(4):1357–63. 10.1210/jc.2005-2430 16434451

[127] Westerveld HE Hoogendoon M de Jong AWF Goverde AJ Fauser BCJM Dallinga-Thie GM. Cadiometabolic abnormalities in the polycystic ovary syndrome: pharmacotherapeutic insights. Pharmacol Ther (2008) 1 19:223–41. 10.1016/j.pharmthera.2008.04.009 18602948

[128] Diamanti-Kandarakis E Kandaraki E Christakou C Panidis D. The effect of pharmaceutical intervention on lipid profile in polycystic ovary sydrome. Obes Rev (2009) 10:431–41. 10.1111/j.1467-789X.2009.00588.x 19413702

[129] Dokras A Jagasia DH Maifeld M Sinkey CA VanVoorhis BJ Haynes WG. Obesity/insulin resistance but not hyperandrogenism is an important mediator of vascular dysfunction in women with PCOS. Fertil Steril (2006) 86:1702–9. 10.1016/j.fertnstert.2006.05.038 17067587

[130] Talbott EO Zborowski J Rager J Stragand JR. Is there an independent effect of polycystic ovary syndrome (PCOS) and menopause on the prevalence of subclinical atherosclerosis in middle aged women. Vasc Halth Risk Manage (2008) 4(2):453–62. 10.2147/VHRM.S1452 PMC2496969 18561521

[131] Talbott EO Guzick DS Sutton-Tyrell McHugh-Pemu KP Zborowski JV Remsberg KE. Evidence for association between polycysic ovary syndrome and premature carotid atherosclerosis in middle-aged women. Arterioscler Thromb Vasc Biol (2000) 20:2414–21. 10.1161/01.ATV.20.11.2414 11073846

[132] Solomon CG Hu FB Dunaif A Rich-Edwards JE Stampfer MJ Willett WC. Menstrual cycle irregularity and the risk for future cardiovascular disease. J Clin Endocrinol Metab (2002) 87:2013–7. 10.1210/jcem.87.5.8471 11994334

[133] Salonen Jukka T Salonen R. Ultrasonographically assessed carotid morphology and the risk of coronary heart disease. Arterioscler Thromb (1991) 11:1245–9. 10.1161/01.ATV.11.5.1245 1911709

[134] Burke GL Evans GW Riley WA Sharrett AR Howard G Barnes RW. Arterial wall thickness is associated with prevalent cardiovascular disease in middle-aged adults: the Atherosclerosis Riskin Communities (ARIC) Study. Stroke (1995) 26:386–91. 10.1161/01.STR.26.3.386 7886711

[135] Bots ML Hoew AW Koudstaal PJ Hofman A. Grobbee DE. Common carotid intima-media thickness and risk of stroke and myocardial infarction: the Rotterdam Study. Circulation (1997) 96:1432–7. 10.1161/01.CIR.96.5.1432 9315528

[136] Chambless LE Folsom AR Clegg LX Sharrett AR Shahar E Nieto FJ. Carotid wall thickness is predictive of incident clinical stroke: the Atherosclerosis Risk in Communities (ARIC) study. Am J Epidemiol (2000) 151:478–87. 10.1093/oxfordjournals.aje.a010233 10707916

[137] Kuller LH Shemanski L Psaty BM Borhani NO Gardin J Haan MN. Subclinical disease as an independent risk factor for cardiovascular disease. Circulation (1995) 92:720–6. 10.1161/01.CIR.92.4.720 7641349

[138] O’Leary DH Polak JF Kronmal RA Manolio TA Burke GL Wolfson SK Jr. Carotid-artery intima and media thickness as a risk factor for myocardial infarction and stroke in older adults. N Engl J Med (1999) 340:14–22. 10.1056/NEJM199901073400103 9878640

[139] Tonstad S Joakimsen O Stensland-Bugge E Leren TP Ose L Russell D. Risk factors related to carotid intima-media thickness and plaque in children with familial hypercholesterolemia and control subjects. Arterioscler Thromb Vasc Biol (1996) 16:984–91. 10.1161/01.ATV.16.8.984 8696963

[140] Chambless LE Heiss G Folsom AR Rosamond W Szkle M Charrett AR. Association of coronary heart disease incidence with carotid arterial wall thickness and major risk factors: the Atherosclerosis Risk in Communities (ARIC) Study, 1987–1993. Am J Epidemiol (1997) 146:483–94. 10.1093/oxfordjournals.aje.a009302 9290509

[141] Folsom AR Eckfeldt JH Weitzman S Jing M Chambless LE Barnes RW. Relation of carotid artery wall thickness to diabetes mellitus, fasting glucose and insulin, body size, and physical activity. Stroke (1994) 25:66–73. 10.1161/01.STR.25.1.66 8266385

[142] Lassila HC Tyrrell KS Matthews KA Wolfson SK Kuller LH. Prevalence and determinants of carotid atherosclerosis in healthy postmenopausal women. Stroke (1997) 28:513–7. 10.1161/01.STR.28.3.513 9056604

[143] Sutton-Tyrrell K Alcorn HG Herzog H Kelsey SF Kuller LH. Morbidity, mortality, and antihypertensive treatment effects by extent of atherosclerosis in older adults with isolated systolic hypertension. Stroke (1995) 26:1319–24. 10.1161/01.str.26.8.1319 7631329

[144] Bonithon-Kopp C Scarabin P-Y Taquet A Touboul P-J Malmejac A Guize L. Risk factors for early carotid atherosclerosis in mddle-aged French women. Arterioscler Thromb (1991) 11:966–72. 10.1161/01.atv.11.4.966 2065047

[145] Dobs AS Nieto FJ Szklo M Barnes R Sharrett AR Ko W-J. Risk factors for popliteal and carotid wall thicknesses in the atherosclerosis risk in communities (ARIC) study. Am J Epidemiol (1999) 150:1055–67. 10.1093/oxfordjournals.aje.a009929 10568620

[146] Folsom AR Wu KK Shahar E Davis CE for the Atherosclerosis Risk in Communities (ARIC) Study Investigators. Association of hemostatic variables with prevalent cardiovascular disease and asymptomatic carotid artery atherosclerosis. Arterioscler Thromb (1993) 13:1829–36. 10.1161/01.atv.13.12.1829 8241104

[147] Meyer ML Malek AM Wild RA Korytkowski MT Talbott EO. Carotid artery intima-media thickness in polycystic ovary syndrome:a systemic review and meta-analysis. Hum Reprod Update (2012) 18(2):122–26. 10.1093/humupd/dmr046 PMC3383099 22108382

[148] Arad Y Spadaro LA Goodman K Newstein D. Guerci AD 2000 Prediction of coronary events with electron beam computed tomography. J Am Coll Cardiol (2000) 36:1253–60. 10.1016/S0735-1097(00)00872-X 11028480

[149] Christian RC Dumesic DA Behrenbeck T Oberg AL Sheedy PF Fitzpatrick LA. Prevalence and predictors of coronary artery calcification in women with polycystic ovary syndrome. J Clin Endocrinol Metab (2003) 88(6):2562–8. 10.1210/jc.2003-030334 12788855

[150] Talbott EO Zborowski JV Rager JR Boudreaux MY Edmundowicz DA Guzick DS. Evidence for an association between metabolic cardiovascular syndrome and coronary and aortic calcification mong women with polycystic ovary syndrome. J Clin Endocrinol Metab (2004) 89(11):5454–61. 10.1210/jc.2003-032237 15531497

[151] Shroff R Kerchner A Maifeld M Van Beek EJ Jagasia D Dokras A. Young obese women with polycystic ovary syndrome have evidence of early coronary atherosclerosis. J Clin Endocrinol Metab (2007) 92(12):4609–14. 10.1210/jc.2007-1343 17848406

[152] Pierard M Tassin A Conotte S Zouaoui Boudjeltia K Legrand A. Sustained Intermittent Hypoxemia Induces Adiponectin Oligomers Redistribution and a Tissue-Specific Modulation of Adiponectin Receptor in Mice. Front Physiol (2019) 10:68. 10.3389/fphys.2019.00068 30800074 PMC6376175

[153] Anuurad E Tracy RP Pearson TA Kim K Berglund L. Synergistic role of inflammation and insulin resistance as coronary artery disease risk factors in African Americans and Caucasians. Atherosclerosis (2009) 205:290–5. 10.1016/j.atherosclerosis.2008.11.028 PMC2700183 19135196

[154] Repaci A Gambineri A Pasquali R. The role of low-grade inflammation in the polycystic ovary syndrome. Mol Cell Endocrinol (2011) 335(1):30–41. 10.1016/j.mce.2010.08.002 20708064

[155] Agarwal A Gupta S Sharma RK. Role of oxidative stress in female reproduction. Reprod Biol Endocrinol RB&E (2005) 3:28–8. 10.1186/1477-7827-3-28 PMC1215514 16018814

[156] Forman HJ Fukuto JM Torres M. Redox signaling: thiol chemistry defines which reactive oxygen and nitrogen species can act as second messengers. Am J Physiol Cell Physiol (2004) 287:C246–256. 10.1152/ajpcell.00516.2003 15238356

[157] Fujii H Nakai K Fukagawa M. Role of Oxidative Stress and Indoxyl Sulfate in Progression of Cardiovascular Disease in Chronic Kidney Disease. Ther Apheresis Dialysis (2011) 15:125–8. 10.1111/j.1744-9987.2010.00883.x 21426501

[158] Biondi R Brancorsini S Poli G Egidi MG Capodicasa E Bottiglieri L. Detection and scavenging of hydroxyl radical via D-phenylalanine hydroxylation in human fluids. Talanta (2018) 181:172–81. 10.1016/j.talanta.2017.12.084 29426497

[159] Valko M Rhodes CJ Moncol J Izakovic M Mazur M. Free radicals, metals and antioxidants in oxidative stress-induced cancer. Chem Biol Interact (2006) 160:1–40. 10.1016/j.cbi.2005.12.009 16430879

[160] Sayre LM Smith MA Perry G. Chemistry and biochemistry of oxidative stress in neurodegenerative disease. Curr Med Chem (2001) 8:721–38. 10.2174/0929867013372922 11375746

[161] Birben E Sahiner UM Sackesen C Erzurum S Kalayci O. Oxidative stress and antioxidant defense. World Allergy Organ J (2012) 5:9–19. 10.1097/WOX.0b013e3182439613 23268465 PMC3488923

[162] Patel RP McAndrew J Sellak H White CR Jo H Freeman BA. Biological aspects of reactive nitrogen species. Biochim Biophys Acta (1999) 1411:385–400. 10.1016/s0005-2728(99)00028-6 10320671

[163] Halliwell B Gutteridge JM Cross CE. Free radicals, antioxidants, and human disease: where are we now? J Lab Clin Med (1992) 119:598–620. 10.1016/S0140-6736(94)92211-X 1593209

[164] Pierce JD Cackler AB Arnett MG. Why should you care about free radicals? Rn (2004) 67:38–42; quiz 43. 14979192

[165] Szczepańska M Koźlik J Skrzypczak J Mikołajczyk M. Oxidative stress may be a piece in the endometriosis puzzle. Fertil Steril (2003) 79:1288–93. 10.1016/s0015-0282(03)00266-8 12798872

[166] Van Langendonckt A Casanas-Roux F Donnez J. Oxidative stress and peritoneal endometriosis. Fertil Steril (2002) 77:861–70. 10.1016/s0015-0282(02)02959-x 12009336

[167] Attaran M Pasqualotto E Falcone T Goldberg JM Miller KF Agarwal A. The effect of follicular fluid reactive oxygen species on the outcome of in vitro fertilization. Int J Fertil Women’s Med (2000) 45:314–20. 11092702

[168] Phaniendra A Jestadi DB Periyasamy L. Free radicals: properties, sources, targets, and their implication in various diseases. Indian J Clin Biochem IJCB (2015) 30:11–26. 10.1007/s12291-014-0446-0 25646037 PMC4310837

[169] García-Sánchez A Miranda-Díaz AG Cardona-Muñoz EG. The Role of Oxidative Stress in Physiopathology and Pharmacological Treatment with Pro- and Antioxidant Properties in Chronic Diseases. Oxid Med Cell Longevity (2020) 2020:2082145–2082145. 10.1155/2020/2082145 PMC7396016 32774665

[170] Papalou O Victor VM Diamanti-Kandarakis E. Oxidative Stress in Polycystic Ovary Syndrome. Curr Pharm Design (2016) 22:2709–22. 10.2174/1381612822666160216151852 26881435

[171] Wallace DC. Mitochondrial DNA mutations in disease and aging. Environ Mol Mutagen (2010) 51:440–50. 10.1002/em.20586 20544884

[172] Khashchenko E Vysokikh M Uvarova E Krechetova L Vtorushina V Ivanets T. Activation of Systemic Inflammation and Oxidative Stress in Adolescent Girls with Polycystic Ovary Syndrome in Combination with Metabolic Disorders and Excessive Body Weight. J Clin Med (2020) 9(5):1399. 10.3390/jcm9051399 PMC7291245 32397375

[173] Sathyapalan T Atkin SL. Mediators of inflammation in polycystic ovary syndrome in relation to adiposity. Mediators Inflamm (2010) 2010:758656. 10.1155/2010/758656 20396393 PMC2852606

[174] Repaci A Gambineri A Pasquali R. The role of low-grade inflammation in the polycystic ovary syndrome. Mol Cell Endocrinol (2011) 335:30–41. 10.1016/j.mce.2010.08.002 20708064

[175] Asemi Z Samimi M Tabassi Z Shakeri H Sabihi SS Esmaillzadeh A. Effects of DASH diet on lipid profiles and biomarkers of oxidative stress in overweight and obese women with polycystic ovary syndrome: a randomized clinical trial. Nutr (Burbank Los Angeles County Calif) (2014) 30:1287–93. 10.1016/j.nut.2014.03.008 25194966

[176] Kazemi M Jarrett BY Vanden Brink H Lin AW. Obesity, Insulin Resistance, and Hyperandrogenism Mediate the Link between Poor Diet Quality and Ovarian Dysmorphology in Reproductive-Aged Women. Nutrients (2020) 12(7):1953. 10.3390/nu12071953 PMC7399845 32629978

[177] Tosatti JAG Alves MT Cândido AL Reis FM Araújo VE Gomes KB. Influence of n-3 fatty acid supplementation on inflammatory and oxidative stress markers in patients with polycystic ovary syndrome: a systematic review and meta-analysis. Br J Nutr (2020) 17:1–12. 10.1017/s0007114520003207 32799935

[178] Talbott E Guzick D Clerici A Berga S Detre K Weimer K. Coronary heart disease risk factors in women with polycystic ovary syndrome. Arterioscler Thromb Vasc Biol (1995) 15:821–6. 10.1161/01.atv.15.7.821 7600112

[179] Sulaiman MA Al-Farsi YM Al-Khaduri MM Saleh J Waly MI. Polycystic ovarian syndrome is linked to increased oxidative stress in Omani women. Int J Womens Health (2018) 10:763–71. 10.2147/IJWH.S166461 PMC6276615 30568513

[180] Hyderali BN Mala K. Oxidative stress and cardiovascular complications in polycystic ovarian syndrome. Eur J Obstetr Gynecol Reprod Biol (2015) 191:15–22. 10.1016/j.ejogrb.2015.05.005 26066290

[181] Deanfield JE Halcox JP Rabelink TJ. Endothelial function and dysfunction: testing and clinical relevance. Circulation (2007) 115:1285–95. 10.1161/circulationaha.106.652859 17353456

[182] Spritzer PM Lecke SB Satler F Morsch DM. Adipose tissue dysfunction, adipokines, and low-grade chronic inflammation in polycystic ovary syndrome. Reprod (Cambridge England) (2015) 149:R219–27. 10.1530/rep-14-0435 25628442

[183] Kelly CC Lyall H Petrie JR Gould GW Connell JM Sattar N. Low grade chronic inflammation in women with polycystic ovarian syndrome. J Clin Endocrinol Metab (2001) 86:2453–5. 10.1210/jcem.86.6.7580 11397838

[184] Escobar-Morreale HF Luque-Ramírez M González F. Circulating inflammatory markers in polycystic ovary syndrome: a systematic review and metaanalysis. Fertil Steril (2011) 95:1048–58.e1041-1042. 10.1016/j.fertnstert.2010.11.036 PMC3079565 21168133

[185] Sathyapalan T Kilpatrick ES Coady AM Atkin SL. The effect of atorvastatin in patients with polycystic ovary syndrome: a randomized double-blind placebo-controlled study. J Clin Endocrinol Metab (2009) 94:103–8. 10.1210/jc.2008-1750 18940877

[186] Webb MA Mani H Robertson SJ Waller HL Webb DR Edwardson CL. Moderate increases in daily step count are associated with reduced IL6 and CRP in women with PCOS. Endocrine Connect (2018) 7:1442–7. 10.1530/ec-18-0438 PMC6301194 30475222

[187] Herman R Jensterle Sever M Janez A Dolzan V. Interplay between Oxidative Stress and Chronic Inflammation in PCOS: The Role of Genetic Variability in PCOS Risk and Treatment Responses. London, UK: IntechOpen (2019). 10.5772/intechopen.88698

[188] Murri M Luque-Ramírez M Insenser M Ojeda-Ojeda M Escobar-Morreale HF. Circulating markers of oxidative stress and polycystic ovary syndrome (PCOS): a systematic review and meta-analysis. Hum Reprod Update (2013) 19:268–88. 10.1093/humupd/dms059 23303572

[189] Tyagi N Sedoris KC Steed M Ovechkin AV Moshal KS Tyagi SC. Mechanisms of homocysteine-induced oxidative stress. Am J Physiol Heart Circ Physiol (2005) 289:H2649–56. 10.1152/ajpheart.00548.2005 16085680

[190] Shenoy V Mehendale V Prabhu K Shetty R Rao P. Correlation of serum homocysteine levels with the severity of coronary artery disease. Indian J Clin Biochem IJCB (2014) 29:339–44. 10.1007/s12291-013-0373-5 PMC4062675 24966483

[191] Veeranna V Zalawadiya SK Niraj A Pradhan J Ference B Burack RC. Homocysteine and reclassification of cardiovascular disease risk. J Am Coll Cardiol (2011) 58:1025–33. 10.1016/j.jacc.2011.05.028 21867837

[192] Mangge H Becker K Fuchs D Gostner JM. Antioxidants, inflammation and cardiovascular disease. World J Cardiol (2014) 6:462–77. 10.4330/wjc.v6.i6.462 PMC4072837 24976919

[193] Pang X Liu J Zhao J Mao J Zhang X Feng L. Homocysteine induces the expression of C-reactive protein via NMDAr-ROS-MAPK-NF-κB signal pathway in rat vascular smooth muscle cells. Atherosclerosis (2014) 236:73–81. 10.1016/j.atherosclerosis.2014.06.021 25016361

[194] Zhang S Bai YY Luo LM Xiao WK Wu HM Ye P. Association between serum homocysteine and arterial stiffness in elderly: a community-based study. J Geriatric Cardiol JGC (2014) 11:32–8. 10.3969/j.issn.1671-5411.2014.01.007 PMC3981981 24748879

[195] Faeh D Chiolero A Paccaud F. Homocysteine as a risk factor for cardiovascular disease: should we (still) worry about? Swiss Med Weekly (2006) 136:745–56. 2006/47/smw-11283 10.4414/smw.2006.11283 17225194

[196] Sathyapalan T David R Gooderham NJ Atkin SL. Increased expression of circulating miRNA-93 in women with polycystic ovary syndrome may represent a novel, non-invasive biomarker for diagnosis. Sci Rep (2015) 5:16890. 10.1038/srep16890 26582398 PMC4652283

[197] Chen B Xu P Wang J Zhang C. The role of MiRNA in polycystic ovary syndrome (PCOS). Gene (2019) 706:91–6. 10.1016/j.gene.2019.04.082 31054362

[198] Lu H Buchan R Cook S Lu H Buchan RJ Cook SA. MicroRNA-223 regulates Glut4 expression and cardiomyocyte glucose metabolism. Cardiovasc Res 86: 410-420. Cardiovasc Res (2010) 86:410–20. 10.1093/cvr/cvq010 20080987

[199] Butler AE Ramachandran V Hayat S Dargham SR Cunningham TK Benurwar M. Expression of microRNA in follicular fluid in women with and without PCOS. Sci Rep (2019) 9:16306. 10.1038/s41598-019-52856-5 31705013 PMC6841741

[200] Deswal R Dang AS. Dissecting the role of micro-RNAs as a diagnostic marker for polycystic ovary syndrome: a systematic review and meta-analysis. Fertil Steril (2020) 113:661–9.e662. 10.1016/j.fertnstert.2019.11.001 32192599

[201] Macfarlane L-A Murphy PR. MicroRNA: Biogenesis, Function and Role in Cancer. Curr Genomics (2010) 11:537–61. 10.2174/138920210793175895 PMC3048316 21532838

